# Emerging Trends in Antimicrobial Resistance in Polar Aquatic Ecosystems

**DOI:** 10.3390/antibiotics14040394

**Published:** 2025-04-10

**Authors:** Melissa Bisaccia, Francesca Berini, Flavia Marinelli, Elisa Binda

**Affiliations:** 1 Department of Biotechnology and Life Sciences (DBSV), University of Insubria, 21100 Varese, Italy; melissa.bisaccia@uninsubria.it (M.B.); f.berini@uninsubria.it (F.B.); elisa.binda@uninsubria.it (E.B.); 2 Climate Change Research Center (CCRC), University of Insubria, 22100 Como, Italy

**Keywords:** antimicrobial resistance, antibiotic resistance genes, antibiotics, Antarctica, Arctic, extreme environment, water, climate change

## Abstract

The global spread of antimicrobial resistance (AMR) threatens to plummet society back to the pre-antibiotic era through a resurgence of common everyday infections’ morbidity. Thus, studies investigating antibiotic resistance genes (ARGs) and antibiotic-resistant bacteria (ARB) in urban, agricultural, and clinical settings, as well as in extreme environments, have become increasingly relevant in the One Health perspective. Since the Antarctic and Arctic regions are considered amongst the few remaining pristine environments on Earth, the characterization of their native resistome appears to be of the utmost importance to understand whether and how it is evolving as a result of anthropogenic activities and climate change. In the present review, we report on the phenotypic (e.g., disk diffusion test) and genotypic (e.g., PCR, metagenomics) approaches used to study AMR in the aquatic environment of polar regions, as water represents one of AMR main dissemination routes in nature. Their advantages and limits are described, and the emerging trends resulting from the analysis of ARB and ARGs diffusion in polar waters discussed. The resistome detected in these extreme environments appears to be mostly comparable to those from more anthropized areas, with the predominance of tetracycline, β-lactam, and sulfonamide resistance (and related ARGs). Indeed, AMR is, in all cases, more consistently highlighted in sites impacted by human and wildlife activities with respect to more pristine ones. Surprisingly, aminoglycoside and fluroquinolone determinants seem to have an even higher incidence in the Antarctic and Arctic aquatic environment compared to that from other areas of the world, corroborating the need for a more thorough AMR surveillance in these regions.

## 1. Introduction

Antimicrobial resistance (AMR) is globally acknowledged as a major emerging health challenge, which could plummet society back to the pre-antibiotic era, when even common everyday infections and routine surgical procedures could easily turn life-threatening. A recent comprehensive survey about global AMR revealed how, in 2021, 4.71 million and 1.14 million deaths could be either associated with or attributed to it, respectively [1,2]. It was further estimated that the number of AMR-related deaths could rise as high as 50 million per year by 2050, with substantial economic consequences [3]. It is therefore not surprising that the number of literature reports focusing on AMR has been constantly increasing in recent years, in particular concerning its occurrence in urban, agricultural, and clinical settings, where it may pose greater risks to human health [4,5,6,7,8].

On the other hand, the Antarctic and Arctic regions are considered amongst the few remaining pristine environments on Earth. The characterization of their native resistome appears to be of the utmost importance to understand whether and how it is evolving as a result of anthropogenic activities and climate change, and what consequences this could have on a global level. Studying the polar resistome may help identify ancient antibiotic resistance genes (ARGs) from the pre-antibiotic era and elucidate their origin and mobilization, providing a baseline to reconstruct the environmental impact of the intensive use and overuse of antibiotics [9,10,11,12,13]. At the same time, the presence of contaminants such as heavy metals, pharmaceuticals and other persistent compounds is increasingly reported even in these most remote areas of the planet [14,15,16,17,18], along with that of non-autochthonous microorganisms and ARGs [19,20,21]. This phenomenon is likely correlated to an increase in anthropogenic activities for tourism or research purposes and to wastewater disposal practices near the polar research stations, and it is cause for concern. In fact, it could contribute to AMR dissemination within local microbial communities by strengthening selective pressure through exposition to sub-inhibitory concentrations of xenobiotics and favoring horizontal gene transfer (HGT) mechanisms [22,23,24,25,26]. Finally, the warming effects of climate changes are causing permafrost thawing and glacier ice melting, likely further reactivating and mobilizing ancient ARGs, antibiotic-resistant bacteria (ARB) and viruses, which might represent a new threat to human health if released and propagated to other regions [27,28,29,30]. Indeed, warming temperatures, increased humidity, and variations in precipitation patterns are causing significant alterations in the structure and dynamics of native microbial communities in the polar regions. These may actually result in an accelerated evolution of pathogenic microorganisms and their resistance mechanisms, favoring those microbial communities that rapidly adapt to shifting environmental conditions [31,32].

Most of the studies on the polar resistome to date have explored the ARG diversity in Antarctic and Arctic soils [11,12,20,29,30,31,32,33,34], with only a limited fraction focusing on the aquatic environment. However, water is one of the main bacterial habitats on Earth, actively involved in the dissemination of microorganisms in nature. Water has already been proven as a significant reservoir of AMR in urban settings, as up to 80% of the antimicrobials administered to humans and/or animals can then be excreted in a still-active form through urine and feces. From there, they can reach the aquatic environment following various routes, from wastewater discharge to animal farms and aquaculture run-off. Antibiotic residues can thus enter both surface and groundwater, favoring the acquisition and maintenance of ARGs in environmental bacteria and posing a risk to human health through direct exposure in the food chain (i.e., consumption of aquatic organisms), in drinking water, and for recreational purposes (e.g., swimming), or because of the possibility of ARGs transmission to clinical pathogens [35,36,37,38]. Moreover, ARGs have also been found enriched in various remote extreme aquatic environments, such as high-altitude lakes, salt lakes, underwater caves, and other deep-sea ecosystems [39,40,41,42,43]. An increase in ARG content in downstream habitats (e.g., rivers, lakes, sea) as a consequence of glacier ice melting has been reported as well [27].

The aim of this review is therefore to analyze the state of the art on AMR studies in Antarctic and Arctic water samples, with a particular focus on the characteristics of the environmental samples being analyzed and the approaches selected for this purpose. The goal is dual: to provide researchers with a guide on the various methodologies that could be used from the very beginning of the process (i.e., selection of the sampling sites and sampling protocols) to the last phases (data interpretation), as well as to set a baseline for the standardization of such approaches. To date, thirty-nine papers have been found in the literature investigating AMR in water samples (seawater, fresh waters, wastewater, ice melting water, etc.) from the polar regions over a fourteen-year-period from January 2010 to January 2025 (using antibiotic resistance + polar/Arctic/Antarctic + water/marine/sea/seawater/ocean/lake/pond/wastewater/ice as research strings in PubMed). The environmental resistome in these works was investigated by applying different methods, which can be broadly classified in two main categories: phenotypic approaches and genotypic ones. The first category requires culture-dependent strain isolation from environmental samples and allows to directly test antibiotics’ effects on bacterial growth [44,45]. The latter focuses on the molecular detection of the genetic determinants for AMR (i.e., ARGs) and can be applied both directly on the total extracted environmental DNA or on DNA from specific isolated strains [46,47]. When combined, the two types of approaches concur to obtain a more comprehensive picture of the resistome under investigation, surpassing the limits which are intrinsic to each single protocol. Sensitivity, specificity, coverage, cost, and applicability of the different techniques available to study AMR in polar environments are summarized in Table 1, with further details on their respective pros and cons and examples of how they were applied in the polar regions provided in the following paragraphs. For further comparisons of the methods listed here for studying AMR in the One Health perspective, readers can refer to [48,49,50,51].

## 2. Sampling Sites

**Figure 1:**
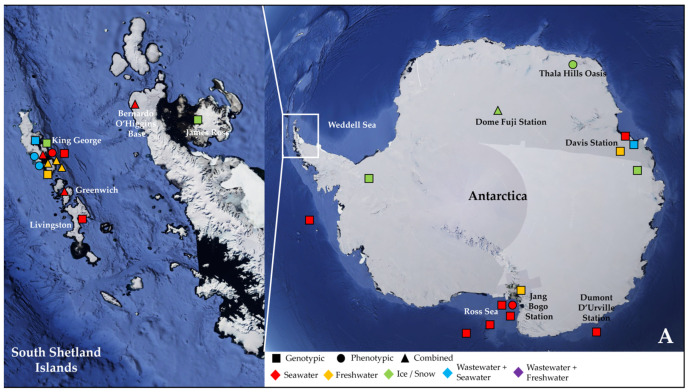

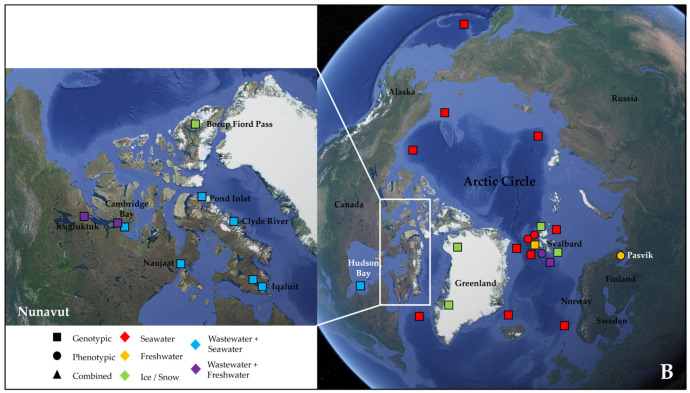
Map showing the geographical sites in Antarctica (with a focus on the South Shetland Archipelago) (**A**) and within the Arctic Circle (with a focus on the region of Nunavut in the Canadian Arctic) (**B**), where samples were collected and studied in the papers reviewed in this work (see Table 2). Sampling points in the sea were localized according to the research cruise routes. The studies were classified according to the type of environmental samples collected (color, either seawater, freshwater, ice/snow, or combinations with wastewater) and to the type of approach chosen for investigating AMR (symbol, either genotypic, phenotypic, or a combination of the two).

More than half of these papers (23) dealt with the Antarctic water environment [13,21,52,53,54,55,56,57,58,59,60,61,62,63,64,65,66,67,68,69,70,71,72] (Figure 1A), with several studies localized in King George Island from the archipelago of the South Shetlands, one of the areas with the highest concentration of scientific research stations in the world [58,59,61,62,63,64,67,68,69,72] (Figure 1A). Fourteen papers investigated water samples collected in regions within the Arctic Circle, which includes territories from Norway, Sweden, Finland, the United States (Alaska), Canada (Yukon, Northwest Territories, Nunavut), Denmark (Greenland), and Iceland (Grimsey) [27,28,73,74,75,76,77,78,79,80,81,82,83,84] (Figure 1B), focusing mainly on the isolated rural communities of Nunavut [74,76,77,83] or on the Svalbard Archipelago [27,73,75,78,80,81,82,84] (Figure 1B). Only two papers actually investigated both the North and South Pole together [85,86] (Table 2). 

For instance, an extensive metagenomic study of the Arctic and Antarctic marine microbiota from thirty-nine Arctic sites and twenty-one Antarctic ones, comprising different sampling depths from 0 m to 4000 m, was conducted by Cao et al. (2020) [85]. All main research activities in Antarctica are governed by the Antarctic Treaty System (ATS), established in 1959, which bans mineral resource activities, except for scientific research, mandates environmental impact assessments for all activities, and sets guidelines for waste management and marine pollution prevention [87]. Based on the ATS, special permissions are required for taking native mammals or birds, entering specially protected areas, or introducing species; therefore, this must always be taken into consideration during the planning of AMR studies in Antarctica. The Scientific Committee on Antarctic Research (SCAR) Code of Conduct provides specific guidance for scientists undertaking terrestrial scientific field research in Antarctica, ensuring that human presence will have as little impact as possible and that the Antarctic environment is protected for future generations [87]. On the contrary, the Arctic lacks a single comprehensive treaty, with governance referring to national laws of the eight Arctic states and international agreements. However, the Arctic Council, the leading intergovernmental forum for cooperation, coordination and interaction among the Arctic States, promotes several initiatives related to the sustainable development and environmental protection of the Arctic. As such, permits are still most often required for fieldwork, sample collection, and the use of certain equipment [88]. Of particular relevance for AMR studies in the Arctic aquatic environment are the activities from the PAME, the Arctic Council working group for the Protection of the Arctic Marine Environment [89].

The vast majority of the papers (25) analyzed seawater samples [21,27,53,54,55,57,58,59,60,62,65,66,67,68,69,71,72,73,74,77,80,81,82,83,84,85], with a more limited number (11) devoted to freshwater environments (e.g., lakes and rivers) [56,61,63,64,71,72,75,76,78,79,80] (Figure 1A,B). Six studies reported on ice and snow samples [13,28,68,70,80,86], and one research investigated temporary meltwater ponds formed in Antarctica in summer as a result of ice and snow melting [52]. In addition, Caruso et al. (2024), González-Pleiter et al. (2021), and Laganà et al. (2019) studied AMR amongst the plastic microbial colonizers forming the plastisphere, as it has been proposed that plastics could enhance AMR transmission in water due to their ability to adsorb various xenobiotics and to act as substrates for microbial adhesion and biofilm formation [54,62,75]. In particular, González-Pleiter et al. (2021) evaluated bacteria associated with biodegradable (poly-3-hydroxybutyrate) and non-biodegradable (polyethylene, PE) microplastics in an Arctic lake [75]. Caruso et al. (2024) and Laganà et al. (2019) focused instead on the Antarctic marine environment, studying bacteria growing on polyvinyl-chloride and PE panels submerged in Terra Nova Bay, or on a polystyrene macro-sized block along the coast of Maxwell Bay, respectively [54,62]. Other studies analyzed sediments or soil samples from the shores of the investigated aquatic environments (or cryoconite particles from glacier environments) to evaluate whether the corresponding microbial communities showed similar dynamics and resistome to those from water or displayed instead significant differences [21,27,28,65,70,75,77,78,79,80,81,82,84] (Table 2). Some papers also included aquatic animals, such as marine invertebrates, shellfish, and fishes, or fecal samples from seals, migratory birds, and penguins, which could play an active role in disseminating ARGs across environments and into the food chain [21,53,58,65,67] (Table 2).

In a significant percentage (26%) of the papers, wastewater was collected and analyzed in combination with either seawater [21,59,67,69,74,77,80,83] or freshwater [76,78,80] from nearby sites (Figure 1A,B and Table 2). Wastewater treatment systems (WWTSs) are generally unable to completely eliminate ARB and ARGs, managing in some cases to lower their load, while, in others, a concentration effect was actually observed at the end of the process [90,91]. Thus, studies sampling in parallel wastewater from WWTSs and water from the receiving aquatic environment are common practice to evaluate their impact and the effect of released nutrients and contaminants on the local bacterial community and their ARG reservoir. Moreover, in polar regions, WWTSs face several environmental and logistical challenges, and are therefore subjected to particular specific regulations. In Antarctica, the Protocol on Environmental Protection to the Antarctic Treaty (Madrid Protocol, Annex III) states that sewage and domestic liquid wastes can be directly discharged into the surrounding marine environment, provided that the conditions allow for rapid initial dilution and dispersal [21,87]. In the Canadian Arctic (where most rural communities do not possess piped water or wastewater infrastructures), waste stabilization ponds (WSPs) are the main passive system for treating municipal wastes, with the wastewater being held for an average of 200–250 days, followed by an annual decant into the marine environment at the end of the summer [83,89]. Consequently, several different samplings at the chosen site were performed in the various studies to account for seasonal and yearly variations as well as to assess other parameters, such as distance from the outfall, time prior or after discharge, or sampling depth. Stark et al. (2016) investigated thirty sites, whose distance from the outfall of Davis Station (Vestfold Hills, East Antarctica) WWTS ranged from hundreds of meters to over 10 km (straight line distance) [21], while Hernández et al. (2019) collected samples from sites evenly distributed around the sewage outfalls of three Antarctic stations on Fildes (Maxwell) Bay (King George Island). Seawater samples were collected from the sea surface, on the coastline and in the open sea, at distances of 0, 10, 15, 25, 50, 150, 350, and 650 m from sewage outfalls [59]. Finally, Szopińska et al. (2022) assessed the degree of wastewater dispersion on the western shore of Admiralty Bay in the vicinity of Arctowski Station (King George Island) by sampling before and immediately after discharge, and after 1, 2, 24, 48, and 96 h [69].

## 3. Sampling Protocols

The samples were generally collected manually, using Niskin bottles, which are plastic cylinders with stoppers at each end, allowing for a complete seal of the bottle. This device was used to sample water at the desired depths before transferring it into appropriately sterilized containers. The samples were then stored either in ice, before being quickly processed in 24–48 h, or at −20 °C or −80 °C for longer periods of time. During sampling, several parameters were often measured as water quality indicators. The most common were temperature, pH, dissolved oxygen, salinity, conductivity, along with ammonia and nitrate content [52,59,72,77,78,79,81,82,83,84]. Three studies carried out more detailed analyses on the collected seawater or wastewater samples, using ultra-high-performance liquid chromatography–mass spectrometry to detect the levels of antibiotics, anti-convulsant drugs, antidepressants, β-blockers, non-steroidal inflammatory drugs, pharmaceutical and personal care products, and other emerging contaminants which can quite commonly be found in wastewater and could have a role in selecting or co-selecting for AMR [59,69,74].

After being collected, water samples were subjected to one or more filtering steps, which served multiple purposes: (i) to remove coarse particles and other macro-contaminants that may interfere with the following analyses (e.g., cause enzyme inhibition during polymerase chain reaction (PCR)); (ii) to separate, select, and retain different size microorganisms (e.g., viruses, free-living bacteria, aggregated bacteria); and (iii) to concentrate samples [13,21,53,55,58,59,60,65,67,68,69,72,73,74,75,76,77,78,80,83,84,85,86]. In most cases, filtration involved a single step using membrane filters in either cellulose-acetate, polycarbonate, or nylon and with a porosity of either 0.22 µm [68,69,72,73,75,76,80,84,85] or 0.45 µm [21,65,77,78,80,83] but some more complex protocols were also applied, especially when virus, phages, or bacteria had to be retained. Blanco-Picazo et al. (2020) recovered phage particles from seawater samples by successively filtering them with 5 µm Swinnex^®^ filter holders (Millipore, Merck KGaA, Darmstadt, Germany), 3 µm mixed cellulose ester membranes (Merck KGaA), and 0.2 µm Isopore^TM^ polycarbonate membranes (Merck KGaA), before performing a chemical flocculation step with iron chloride and a final filtration through 1.2 µm Isopore^TM^ membranes (Merck KGaA) [53]. Cuadrat et al. (2020) applied a single or a combination of membrane filters with pore sizes of 0.1, 0.2, 0.45, 0.8, 1.6, and 3 µm to retain different size fractions enriched for viruses, giant viruses, and prokaryotes [55]. Ice core and snow samples were usually melted at room temperature and then filtered after careful removal of the first outer layers (10–20 mm), containing potential surface contaminants [13,80,86]. The filters could then serve to isolate microorganisms through culture-dependent (selective) methods [21,58,59,65,67,78] but were more often directly used to extract DNA for molecular ARG detection [13,53,55,60,68,69,72,73,74,75,76,77,80,83,84,85,86].

## 4. Culture-Dependent Isolation and Phenotypic Approaches

In seventeen studies (44% of the total 39 articles covered in this review) [21,52,54,57,58,59,61,62,63,64,65,66,67,78,79,81,82], culture-dependent methods were used to isolate various types of microorganisms from the collected Arctic and/or Antarctic water samples and to evaluate their AMR pattern (Table 2). In six of these articles [21,58,59,65,67,78], filters were directly employed for the microbial isolation procedure. For instance, Power et al. (2016) and Stark et al. (2016) applied the US EPA Method 1604 (membrane filtration method) to simultaneously isolate and enumerate both total coliforms (TC) and *Escherichia coli* [21,65,92]. The method consisted of a filtering step through a 0.45 μm pore size cellulose ester membrane filter that retained the bacteria present in the sample and was then placed on MI agar (Merck KGaA) plates. After 24 h of incubation at 35 °C, the colonies were identified based on their color, which depends on the breakdown of the fluorogen 4-methylumbelliferyl-β-d-galactopyranoside by the β-galactosidase produced by TC or of the chromogen indoxyl-β-d-glucuronide by *E. coli* β-glucuronidase [92]. In other cases, water samples were not filtered but used directly or after being further diluted, and isolation was performed through the spread plate methods [52,54,57,61,62,63,64,79,81,82].

Some studies aimed at isolating all possible cultivable heterotrophic bacteria (CHB) and consequently applied non-selective culture conditions, using universal media such as Meat Peptone Agar (Merck KGaA) [52], R2A Agar (Merck KGaA) [61], Nutrient Agar (Merck KGaA) [63], Mueller–Hinton Agar (MHA, Difco, BD, Franklin Lakes, NJ, USA) [64], and Plate Count Agar (Difco, BD) [79], or media recommended for marine microorganisms like Marine Agar (Difco, BD) [54,57,62,79] and Zobell Marine Agar (Himedia, Mumbai, India) [81,82]. The plates were usually incubated at 5–10 °C for up to two or three weeks, as microorganisms isolated from the Arctic and Antarctic regions are expected to be cold-adapted and slow-growing.

On the other hand, efforts were often addressed to isolate only certain types of microorganisms, such as coliforms and *Enterobacteriaceae*, as in the cases of Power et al. (2016) and Stark et al. (2016), reported above [21,65,92]. These bacteria are considered non-autochthonous to polar environments and are often used as indicators of fecal contamination and markers of anthropogenic impact, being possible major vectors in ARGs dissemination. *E. coli* and TC were often detected after 24 h of incubation at 37 °C on the chromogenic selective Chromocult^®^ Coliform Agar (Merck KGaA) medium, where salmon-red colonies indicate coliforms and dark blue to violet *E. coli* [21,58,59,65,67]. For the same purpose, MI Agar (Difco, BD) [21,65], Uriselect4 (Bio-Rad Laboratories Ltd., Hemel Hempstead, UK) [58], Eosin Methylene Blue Agar (Difco, BD) [82], or Lactose Broth (Merck KGaA) [81] were also used. Kalinowska et al. (2021) selectively isolated *Enterococcus* spp. as dark red or maroon colonies using the Slanetz-Bartley *Enterococcus* Selective Agar (Merck KGaA) after 48 h of incubation at 37 °C [78], while Neethu et al. (2015) used Thiocholate Bile Salt Agar (Difco, BD) for enriching *Vibrionaceae* [82]. Following isolation, the strains were streaked to pure culture and, in some cases, further investigated.

In fourteen out of the seventeen papers analyzed in this paragraph (82%), the isolated microorganisms were identified at the species level. This was most commonly achieved through 16S rRNA gene sequencing [52,54,61,78], but various studies applied the polyphasic approach, combining morphological, biochemical, and molecular techniques [62,81,82]. In Otur et al. (2023, 2024) works, novel bacterial species were identified from Antarctica by multilocus sequence analysis (based on sequencing 16S rRNA, *gyrB*, *tuf*, *rpoB*, and *rpoD* genes), biochemical tests, and whole-genome sequencing on the Illumina Novaseq 6000 platform [63,64]. The Illumina platform for whole-genome sequencing was also utilized by Giovannini et al. (2024) to describe a collection of *Gammaproteobacteria* strains of marine origin [57]. Works focusing on the isolation of *E. coli* strains further assigned them to specific phylogenetic groups through quadruplex-PCR, comparing the *chuA*, *yjA*, *TspE4.C2*, and *arpA* gene sequences [21,65], or differentiated them through pulsed-field gel electrophoresis [67].

In thirteen out of the seventeen papers performing microbial isolation (Table 2), the pattern of AMR of the microbial isolates was then investigated through a phenotypic approach (Table 2). The Kirby–Bauer test [93] or disk diffusion test were the most commonly applied phenotypic methods by far (in 92% of the studies) [52,54,58,59,61,62,63,64,67,79,81,82]. In the Kirby–Bauer test, isolates were grown for 48 h on plates of Tryptic Soy Agar (Difco, BD) [62,79], or enriched in Nutrient Broth (Difco, BD) at 37 °C for 24 h [81,82], harvested and then suspended in sterile water to reach a 0.5 McFarland turbidity standard (1.5 × 10^8^ CFU/mL). The inoculum was streaked onto plates of MHA using a sterile cotton swab. Commercially available antibiotic-impregnated disks were used to determine the resistance patterns of the isolates. Plates were incubated for 48 h either at 5 °C [62,79], 15 °C [63], or 18–25 °C [52,54], for 24 h at 20 °C [82] or 37 °C [81], or for 2 h at 4 °C followed by 48 h at 15 °C [64]. For disk diffusion test, the protocol was quite similar to the Kirby–Bauer one, but it specifically followed the guidelines of Clinical and Laboratory Standard Institute (CLSI) [94]. Jara et al. (2020) substituted MHA with R2A Agar in the protocol [61]. Various reference microorganisms were used as susceptibility controls, such as *E. coli* ATCC 25922, *Staphylococcus aureus* ATCC 25923, and *Pseudomonas aeruginosa* ATCC 27853. The inhibition halos produced by the antibiotic disks were then measured with a precision caliber and results were interpreted according to the standards and breakpoints provided by either CLSI [52,59,61,62,63,64,67,79,94] or EUCAST (European Committee on Antibiotic Susceptibility Testing) [52,54,58,62,79,95], or through Himedia charts [81,82], with microorganisms being classified as resistant, intermediate, or sensitive/susceptible. Some authors also calculated the multiple antibiotic resistance (MAR) index for each isolate, using the formula *a*/*b*, where *a* represents the number of antibiotics to which the isolate was resistant and *b* is the total number of antibiotics against which it was tested [54,61,79,81]. Following these guidelines allows to partially standardize protocols and compare the results of different studies, although they have been developed to test AMR in pathogens and clinical isolates, and how significant the provided cut-off values are in evaluating environmental bacteria remains doubtful (Table 1).

In addition to the disk diffusion test, Jara et al. (2020) performed total count of CHB with reduced susceptibility to various antibiotics as well by plating freshwater samples directly on antibiotic-supplemented R2A Agar and incubating the plates at 4 °C for 15 days and at 15 °C for 7 days [61]. Kalinowska et al. (2021) investigated AMR amongst isolated *Enterococcus* spp. by determining the minimum inhibitory concentration (MIC) of commercially available panels of antimicrobial agents, representative of drugs important in treating human enterococcal infection. Microdilution tests were performed with the Phoenix^TM^ Automated Microbiology System (Difco, BD) and *Enterococcus faecalis* ATCC 20212 was used as quality control [78]. The MIC values were set in agreement with epidemiological cut-off value (ECOFF) and with the clinical breakpoints provided by EUCAST [95]. ECOFF is defined based on the normal distribution of MICs for a given bacterial species and provides the upper MIC value for the wild-type population. It takes into consideration that bacteria that have evolved a resistance mechanism as a response to naturally occurring antimicrobial agents usually remain susceptible from the clinical point of view, as antibiotic concentrations used in therapy are different from those naturally occurring in the environment. Thus, using ECOFF in environmental studies allows to distinguish wild-type species lacking the acquired and/or mutational mechanisms of resistance from non-wild ones carrying resistance determinants (Table 1) [78].

Finally, in the works of Hernández et al. (2012), Jara et al. (2020), and Otur et al. (2023, 2024), phenotypic description of AMR was further integrated with genotypic investigations of the corresponding determinants [58,61,63,64], which will be detailed in the following paragraph.

## 5. Genotypic Approaches

Studying AMR distribution in an environment using phenotypic approaches has the limitation that it is relative to only those microbes that can be cultivated under laboratory conditions. Despite the recent advances in innovative cultivation technologies, it has been estimated that only between 0.1% and 1% of the total microorganisms present in an environment are actually cultivable [96,97]. Furthermore, phenotypic susceptibility testing results can be significantly affected by a number of experimental variables, such as antibiotic concentrations, growth media and incubation temperatures. This becomes even more relevant when studying extremophilic microorganisms from the polar regions, which are normally adapted to environmental conditions very different from those reconstructed in the laboratory. Solutions to overcome some of these bottlenecks are presented by the genotypic approaches, where AMR is evaluated by searching for its molecular determinants through various techniques, from PCR to next-generation sequencing (NGS) (Table 1) [46,47,98].

From a genetic point of view, bacterial AMR can either be intrinsic, due to mechanisms normally encoded in the chromosome, or acquired, generally transmitted by HGT [99]. In both cases, there are several different mechanisms which can contribute to it, either singularly or in concert. They can be grouped in three main categories: (i) those that decrease the intracellular concentrations of the antibiotic by hindering its penetration into the cells or actively pumping it out of them through efflux systems; (ii) those that modify the antibiotic target by genetic mutation or post-translational modification, drastically reducing the antibiotic binding affinity; and (iii) those that inactivate the antibiotic itself through enzymatic modifications, such as hydrolysis or acetylation, adenylation and phosphorylation [100,101]. ARGs, which have been identified as involved in any of these mechanisms, are nowadays commonly used as markers for resistance (as listed in Table 3).

Thus, genotypic approaches offer the possibility to gain a more complete perspective on the ARG reservoir present in an environment and, for this reason, have progressively become the preferred method for AMR studies. They were applied in a total of thirty studies (77%) among those covered in this review. In four of these studies, genotypic approaches were applied on already isolated strains [21,57,65,66], whereas in twenty-two studies, they were used to describe the environmental resistome without the need for a cultivation step [13,27,28,53,56,60,68,69,70,71,72,73,74,75,76,77,80,83,84,85,86]. Finally, in four studies (see the end of the previous paragraph), AMR was described both at the level of phenotype and through a genetic-level assessment (Table 2) [58,61,63,64].

### 5.1. Traditional and Quantitative PCR

In four cases out of the thirty involving genotypic approaches, traditional PCR was used to detect ARGs in Antarctic microbial communities (Table 2). In particular, in the works by Power et al. (2016) and Stark et al. (2016), *E. coli* strains isolated from various Antarctic seawater, sediment, and fecal samples were screened by PCR for the presence of the class 1 integron-integrase gene *intI1* [21,65]. Class 1 integrons, derived from Tn402 transposon, are particularly relevant for AMR studies, as they often contain many ARGs and co-select with metal resistance genes. They comprise a highly conserved integrase gene from the tyrosine recombinase family (*IntI1*), a recombination site (*attI*), where the integron-integrase can catalyze insertion or excision of resistance gene cassettes, and a promotor (*Pc*), which drives gene cassette expression [102,103,104,105,106,107]. Humans are colonized with class 1 integrons soon after birth and drive their dissemination via migration and international travel; so, *intI1* can also be used as a marker of anthropogenic impact [105]. Indeed, the *intI1* gene was detected in 20% of the screened Antarctic-sourced *E. coli* strains, with more than half of them being attributed to the B2 phylogenetic group, which comprises strains common in mammals and many extraintestinal pathogens of human and companion animals. Positive strains were further characterized by cassette array PCR [21,65].

Okubo et al. (2019) analyzed Antarctic ice core samples dating back from 1200 up to 2800 years to gain information about ancient ARGs present in the pre-antibiotic era. They first performed whole-genome amplification of the extracted environmental DNA, and then used primers to detect ARGs for β-lactam, aminoglycoside, macrolide, chloramphenicol, fluoroquinolone, and glycopeptide antibiotics. The aminoglycoside phosphotransferase genes *strA* and *strB* and the sulfonamide resistance gene *sul2* were the only ARGs detected. As the authors wanted to verify whether the identified genes could actually confer resistance, the whole sequence of the *sul2*–*strA*–*strB* gene cluster was cloned and the antibiotic susceptibility of the *E. coli* transformant tested, demonstrating the full functionality of these ancient amplified ARGs, which predate the discovery and large-scale use of antibiotics (especially in the case of sulfonamides which are synthetic molecules) [13]. Finally, as previously mentioned, Jara et al. (2020) investigated AMR in Antarctic freshwater samples, integrating phenotypic and genotypic approaches. They isolated all possible CHB, and then characterized their AMR profile by both disk diffusion assay and traditional PCR for ARGs to aminoglycosides, β-lactams, chloramphenicol, fluoroquinolones, and tetracyclines [61].

Quantitative PCR (qPCR) was actually the most frequently applied molecular technique: it was used in 33% (10) of the studies employing genotypic approaches, in the majority of them directly on the DNA extracted from environmental samples (Table 2). In general, it allowed not only to detect the presence or absence of ARGs of interest, but also to quantify their relative abundance, normalizing it to the 16S rRNA gene concentration (Table 1) [108]. González-Pleiter et al. (2021) characterized the polar plastisphere associated with three types of microplastics by evaluating through qPCR the relative abundance of the *sulI* and *ermB* genes, conferring resistance to sulfonamides and macrolides, respectively [75]. Szopińska et al. (2022) applied qPCR to detect class 1, 2, and 3 integron-integrase genes (*intI1*, *intI2*, *intI3*) and sulfonamide resistance genes (*sul1*, *sul2*, *sul3*) to assess the occurrence of emerging contaminants, ARGs and integrons in wastewater and its receiver in the western shore of Admiralty Bay (King George Island) [69]. Chaves-Barquero et al. (2016) examined the efficacy of wastewater treatment in Arctic (Cambridge Bay, NU, Canada), monitoring the release and attenuation of selected nutrients, pharmaceuticals, and ARGs. They extracted DNA from bacterial cells harvested on filters and quantified ARGs through a multiplex qPCR assay, targeting tetracycline and sulfonamide resistance genes [74]. Jang et al. (2022) studied with qPCR the diversity of ARGs towards β-lactams, fluroquinolones, macrolides, sulfonamides, and tetracyclines in seawater samples collected along a transect from the western Pacific Ocean (36° N) to the Southern Ocean (74° S) [60]. Blanco-Picazo et al. (2020) isolated phage particles from Antarctic and Mediterranean seawater samples and local fishes, as bacteriophages seem to play a relevant role in mobilizing and spreading ARGs by transduction. qPCR was applied in this study to amplify β-lactam, sulfonamide, and tetracycline resistance genes on phage-extracted DNA [53]. Neudorf et al. (2017) and Hayward et al. (2018) evaluated through qPCR the occurrence of a broad range of clinically relevant ARGs in wastewater, water and soil samples from tundra wetlands in Arctic Canadian communities [77,83]. Ushida et al. (2010) and Segawa et al. (2013) simultaneously screened by qPCR ice and snow samples from all over the world (e.g., Arctic, Antarctic, Central Asia, North and South America, and Africa) for up to ninety-three different ARGs, covering resistance to aminoglycosides, β-lactams, chloramphenicol, macrolides, tetracyclines, and glycopeptides [70,86]. Finally, Hernández et al. (2012) identified extended-spectrum β-lactamase (ESBL)-producing *E. coli* strains from Antarctic seawater with a cefpodoxime/cefpodoxime + clavulanic acid double-disk test coupled with qPCR [58], while Otur et al. (2024) monitored the gene expression levels of selected ARGs in microbial isolates exposed to antibiotics which they were found phenotypically resistant to [64].

In only one study, the molecular technique applied was digital PCR (dPCR), an evolution of qPCR, which similarly allows for both detection and quantification of ARGs but without the need for standard curves (Table 1) [109]. Bonanno Ferraro et al. (2024) used dPCR to quantify and analyze the distribution of β-lactamases, sulfonamide and tetracycline ARGs in a wide range of ocean water samples, including the Arctic Ocean [73].

### 5.2. Next-Generation Sequencing (Whole-Genome and Shotgun Sequencing)

NGS techniques represent a powerful and increasingly used tool for AMR studies in environmental samples, allowing for the assessment at once of all those ARGs, which have been previously identified and annotated in dedicated databases. Compared to PCR-based techniques, requiring the design of pre-selected specific primers, NGS techniques offer a broader coverage. Nevertheless, they still suffer from some limits, like bias in DNA extraction methods, reaction inhibition by compounds present in complex environmental samples, lack of reference sequences for comparison and low sequence coverage for rare bacterial species. Moreover, NGS is less sensitive than qPCR and dPCR; so, rarer ARGs in samples could go undetected because of insufficient sequencing depth (Table 1) [110].

In ten studies among those covered in this review, ARGs in Arctic and Antarctic water samples were identified by NGS (Table 2), in all cases using Illumina (Solexa) as the sequencing platform of choice [57,63,64,66,68,72,76,80,84,85]. Presta et al. (2016) sequenced the genome of *Flavobacterium* sp. strain TAB 87, isolated from Antarctic seawater near the French Antarctic station Dumont d’Urville, using a paired-end approach. The genome sequence was then screened for secondary-metabolism-related genes with antiSMASH (a specific database for biosynthetic gene clusters annotation) and for ARGs through the Comprehensive Antibiotic Resistance Database (CARD) [66]. Similarly, Giovannini et al. (2024) sequenced the genomes from 58 *Gammaproteobacteria* strains of marine origin belonging to the Collezione Italiana Batteri Antartici (CIBAN) strain collection, screening them for ARGs with the Resistance Antibiotic Gene Identifier (RGI) tool v6.0.3 available in CARD [57]. Otur et al. (2023, 2024) isolated a new species of *Flavobacterium*, named *Flavobacterium aziz-sancarii* [63], and a *Pseudomonas migulae* CAS19 strain [64] from the water of a lake on Ardley Island (Antarctica). The bacterial genomes were sequenced through a pair-end approach and genes encoding for potentially toxic element and/or ARGs were identified against the BacMet (http://bacmet.biomedicine.gu.se; last accessed on 28 February 2025) and MEGARes (https://megares.meglab.org; last accessed on 28 February 2025) databases [63,64]. Interestingly, *F. aziz-sancarii* was found sensitive to clarithromycin, tetracycline, and chloramphenicol by antibiotic susceptibility testing, despite the presence of *mac*, *tet*, and *cat* resistance genes in its genome. This discrepancy could be due to inappropriate incubation time or tested antibiotic concentrations, which were not able to induce the expression of corresponding ARGs, environmental adjustment of gene expression, or by the fact that the detection of a specific gene sequence does not always mean that it is functionally expressed [63].

NGS was used by Cao et al. (2020) to analyze seawater samples collected in the Arctic and Antarctic Oceans, integrating data from the Tara Oceans project (http://ocean-microbiome.embl.de/companion.html; last accessed on 28 February 2025). The project comprehensively studied the structure and function of the global ocean microbiota, but the Arctic and Antarctic regions were greatly underrepresented. The authors, using 16S rRNA gene amplicon sequencing, found that the microbiota from Arctic and Antarctic seawater did share deeper similarities between each other than with that from other oceanic regions [85]. To further explore the functional potential of the polar microbiota, microbial metagenome assembled genomes (MAGs) were reconstructed and polar specific ortholog proteins identified from them and functionally annotated against the COG database (Cluster of Orthologous Genes). Functions related to AMR were found largely enriched in the Arctic specific orthologs [85].

Zhang et al. (2022) made a comparative analysis of the microbiome and resistome from seawater and sediments of Kongsfjorden in the Arctic. Paired-end sequencing was performed and ARGs annotated against CARD version 3.0.9 [84]. The same approach was applied to study 39 water samples (11 from freshwater and 28 from seawater) collected in the ice-free Fildes Region on King George Island [72]. Ren et al. (2024) employed paired-end sequencing to investigate AMR in seven green- and six red-snow samples collected from the Fildes Peninsula (Antarctica), annotating ARGs against CARD as well [68]. Gromala et al. (2021) performed 16S rRNA gene amplicon sequencing and whole-metagenome shotgun sequencing to characterize microbial communities in Arctic WSPs and assess effluent impacts on receiving waters from a microbiological perspective. Metagenomic reads were processed using the ATLAS pipeline (release 2.0.6), with identification and quantification of ARGs carried out using the precomputed 2017 Antibiotic Resistance Factors marker collection from the ShortBRED documentation pages (available at http://huttenhower.sph.harvard.edu/shortbred; last accessed on 28 February 2025). Gene families were grouped manually into larger groups based on information from CARD [76].

Finally, Makowska-Zawierucha et al. (2024) explored AMR in the plasmidome associated with glacial ice and adjacent aquatic environments under different degrees of anthropogenic influence across the Arctic archipelago of Svalbard. Plasmids are particularly relevant in AMR studies because of the central role attributed to them in ARGs dissemination through HGT. However, plasmidomes from environmental samples are difficult to profile. Because of this, the authors performed an enrichment step prior to plasmid isolation. After filtering the collected water environmental samples, they washed the filters with sterile water, centrifuged the solution and inoculated the resulting pellet in either R2A medium or Brain Heart Infusion (BHI) broth, incubating them for 48 h at 22 °C. Plasmid DNA was then extracted and sequenced. The NCBI Antimicrobial Resistance Gene Finder (AMRFinderPlus) version 3.11.11 and its accompanying database were used to find ARGs, while integrons were detected using Integron Finder version 1.5.1 [80].

Another five studies (Table 2) followed a different approach, centered around the bioinformatic analyses of the steadily increasing number of metagenomes and related metadata available in public databases, with the aim to obtain a comprehensive view of the natural context of ARGs, their geographic distribution and their dynamics on a global scale, rather than a local one. Cuadrat et al. (2020) recovered a total of 12 co-assembled metagenomes from various oceanic regions around the world from the datasets of the TARA Oceans project (https://fondationtaraocean.org/en/expedition/tara-oceans/; accessed on 28 February 2025), including the Antarctic province as representative of pristine polar biomes. To screen for ARGs, the deep learning approach DeepARG was developed, taking into account all the ARG categories of three curated and merged databases, that is, the Antibiotic Resistance Genes Database (ARDB), CARD, and UniProt. The results were manually curated, with ARGs checked for misannotations and inconsistencies, and their abundance calculated as reads per kilobase per genome equivalents (RPKG), by counting the number of reads mapped to the ARG, divided by the length of the ARG in kilobase pairs and the number of sequenced genome equivalents. The dissemination potential of ARGs, for example, their presence on easily transmittable mobile genetic elements (MGEs) such as plasmids, was evaluated using PlasFlow version 1.1 [55]. Yang et al. (2019) analyzed metagenomic data from 92 lakes and 30 seas worldwide that were stored in the Sequence Read Archive (SRA, https://www.ncbi.nlm.nih.gov/sra; last accessed on 28 February 2025), with Antarctic samples included in both sets. The data were processed to detect the presence of ARGs against the Structured ARG (SARG) database and of metal resistance genes (MRGs) against the BacMet reference dataset (http://bacmet.biomedicine.gu.se/index.html; last accessed on 28th February 2025). In fact, co-occurrence of MRGs and ARGs in the same MGE can cause co-selection of ARGs as a consequence of heavy metal pollution into the environment [71]. Following a similar bioinformatic approach, Durso et al. (2012) evaluated whether there were differences in the microbiota and ARG distribution between agricultural and non-agricultural samples, including two Antarctic lakes’ metagenomes. To categorize ARGs, the authors used the MG-RAST classification of resistance to antibiotic and toxic compounds (RATC). The SEED database was used to link gene function with microbial taxonomy in order to identify which bacteria were most likely to carry the ARGs [56]. Wang et al. (2024) recovered raw metagenomic data from NCBI and from the European Nucleotide Archive regarding glaciers in the Arctic and on the Tibetan Plateau (cryoconite samples) and their relative downstream aquatic ecosystems (Arctic Ocean, Qinghai Lake, Yangtze River Basin). The aim was to analyze the diversity and distribution of ARGs and viruses in these samples, evaluating, in particular, whether climate change and warming temperatures could play a significant role in driving ARG dissemination. ARGs were annotated against CARD using the RGI tool (https://card.mcmaster.ca/analyze/rgi; last accessed on 28 February 2025) [27]. Likewise, Liu et al. (2023) reconstructed and analyzed the content of virus particles in Arctic and Tibetan Plateau supraglacial cryoconite and ice samples (obtained from SRA), which could be mobilized by glacier melting and act as vectors of ARGs and virulence factors [28].

To conclude, it is worth noting that there is an inherent limit in the so-far described genotypic approaches, that is, the possibility to identify only ARGs, whose functions have been previously elucidated or that share high sequence homology with already characterized ones. A complementary approach would be functional metagenomics, where environmental DNA fragments are cloned into heterologous hosts, which can then be screened for the acquisition of novel AMR phenotypes. Even if functional library construction and screening can be labor-intensive and expression of the cloned sequences in a different host is not always straightforward, functional metagenomics thus far remains one of the few methods which may allow us to discover and identify ARGs belonging to new classes, of which there is no prior knowledge [91]. To our knowledge, however, studies applying this approach to investigate AMR in the polar regions’ aquatic environments have not been published yet.

## 6. Antimicrobial Resistance Trends in Arctic and Antarctic Water Samples

**Figure 2:**
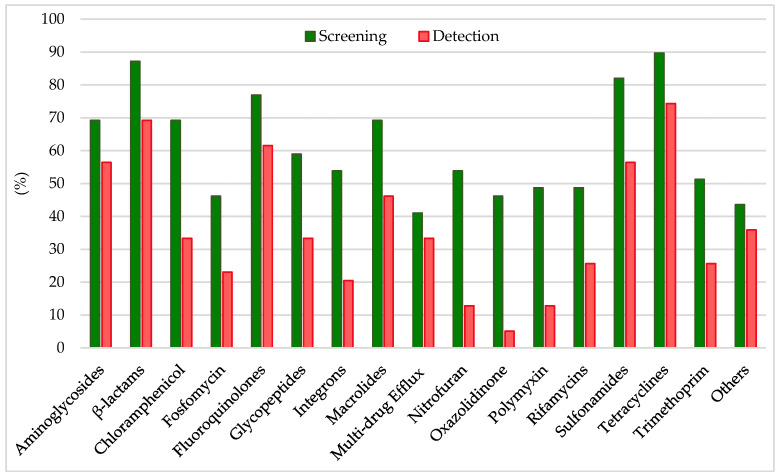
Frequency of screening and detection of resistance against the main antibiotic classes investigated in the AMR studies on Arctic and Antarctic water samples covered in this review, expressed as number of studies in which the antibiotic class is screened or detected per total number of analyzed studies (39).

Analyzing the results from the thirty-nine literature studies on AMR in water samples from the polar regions covered in this review (Table 2), it emerges that resistance to the various antibiotic classes was not equally investigated, either phenotypically or genotypically. In fact, some studies focused only on a small number of antibiotic molecules and/or ARGs, selected by the authors for different reasons (e.g., widespread detection across different types of samples, high worldwide consumption, particular relevance from a clinical point of view), while other papers evaluated much broader panels. The antibiotics classes most frequently screened for resistance in Antarctic and Arctic water samples were tetracyclines, β-lactams, sulfonamides, fluoroquinolones, aminoglycosides, macrolides, chloramphenicol, glycopeptides, and rifamycins, followed by fosfomycin, nitrofuran, polymyxin, and oxazolidinone (Table 3 and Figure 2). The incidence of integrons- and multidrug-efflux-system-related genes was also commonly investigated, exclusively by genetic screening (Table 3 and Figure 2).

### 6.1. Resistance to Tetracyclines

Tetracyclines were first introduced in clinics in the 1940s to treat both Gram-positive and Gram-negative bacterial infections. They have been extensively used for livestock and aquaculture. They inhibit bacterial protein synthesis as they bind inside the decoding center of the 30S ribosomal subunit, interacting with 16S rRNA and blocking aminoacyl-tRNA access to the A-site [111]. At least four different resistance mechanisms to tetracyclines have been described: (i) 16S rRNA mutations that lower tetracyclines’ binding affinity (more common in bacteria with low rRNA copy numbers); (ii) efflux pumps, which actively decrease the antibiotic intracellular concentration (28 distinct classes currently identified; e.g., Tet35, Tet39, Tet41, TetA, TetB, TetC, TetD, TetE, TetG, TetK, TetL, TetZ); (iii) ribosome protection proteins (GTPases, 12 classes identified, e.g., TetM, TetO, TetQ, TetS, TetW); and (iv) monooxygenases, which hydroxylate tetracyclines inactivating them (e.g., TetX) [90,112,113]. Tetracycline resistance was investigated in 90% of the papers on AMR in Antarctic and Arctic water samples and positively detected in 74% of them (Table 3 and Figure 2) by either genotypic or phenotypic approaches. In a study investigating resistome composition along a transect from the western Pacific Ocean to the Southern Ocean, β-lactam and tetracycline ARGs accounted for 88–99% of all the ARGs detected at every station. A slight increase in ARG abundance was detected towards the Antarctic coasts, perhaps due to research and tourism activities or incomplete wastewater treatment practices, with the positive correlation of *tetBP* and *tetZ* with *intI1* further implicating the possible role of HGT [60]. Interestingly, *tetW* was detected in the phage DNA fraction from 70% of seawater samples collected in Antarctica, while it could only be detected in 10% of those from the Mediterranean Sea [53]. Tetracycline resistance genes were also carried by supraglacial viruses from an Arctic glacier [28], suggesting that polar microbial communities may exploit phages for HGT favoring AMR dissemination. In most cases though, *tet* ARGs levels remained quite low [56,57,68,74,77,80,83,84], sometimes just above the limits of detection, indicating that they could simply be part of the environment’s natural background resistome, or that even when introduced by human-related activities, they are rapidly diluted. Phenotypic resistance levels were mainly observed in human-associated bacteria such as *E. coli* strains [58,59,67], which were the focus of several of the studies considered in this review. Resistance amongst autochthonous microorganisms from the polar regions was instead more rarely investigated, despite its importance in understanding whether there is active transmission of ARGs between these two groups of microorganisms. Around 30% of heterotrophic bacteria isolated from the Pasvik River in Norway [79] and 17% of fast-growing bacteria from temporary meltwater bonds in the Thala Hills Oasis region (East Antarctica) [52] were found resistant to tetracycline, while resistance to tigecycline, minocycline, and doxycycline (the more recent semisynthetic derivatives introduced in clinical settings) was practically absent. Taking all these indications together, it is reasonable to state that the frequency of detection of tetracycline resistance in Antarctic and Arctic water samples seems in accordance with literature reports on other water ecosystems, where tetracycline resistance was identified as one of the most common types of AMR across water [114], wastewater [90,115], groundwater [38], lakes and rivers [26,37,116], estuarine and coastal environments [117], as well as in farms and soils [114].

### 6.2. Resistance to β-Lactams

β-lactam antibiotics comprise different classes of molecules that share a common β-lactam ring in their chemical structure: penicillins, cephalosporins, monobactams, and carbapenems [118]. β-lactams account for 65% of all prescriptions for injectable antibiotics in the United States, with cephalosporins representing nearly half of them. All β-lactams are bactericidal and act by inhibiting bacterial cell wall biosynthesis by covalently binding to transpeptidases, also named penicillin-binding proteins (PBPs) [118]. While in Gram-positive bacteria, resistance to β-lactams usually occurs through mutations of the drug target (i.e., PBPs), in Gram-negative bacteria, it depends on the expression of β-lactamases. β-lactamases hydrolytically cleave the β-lactam ring through the action of an active site Serine nucleophile (Ser-β-lactamases), or through activation of water via a Zn^2+^ center (metallo-β-lactamases). They can be further distinguished in ESBL enzymes (conferring resistance to penicillins, cephalosporins, and monobactams, e.g., TEM-, SHV-, CTX-M-, and OXA-type), plasmid-mediated AmpC β-lactamase (AmpC) enzymes (conferring resistance to cephalosporins, e.g., MOX-, CIT-, DHA-, ACC-, and FOX-type), metallo-β-lactamase (MBL) enzymes (conferring resistance to carbapenems and the other β-lactam antibiotics, e.g., NDM-, IMP-, and VIM-type), or Ser-carbapenemase enzymes (conferring resistance to carbapenems and the other β-lactam antibiotics, e.g., KPC-type) [113,119,120]. β-lactam resistance was investigated in 87% of the papers considered in this review and positively detected in 69% of them (Table 3 and Figure 2) by either genotypic or phenotypic approaches. In general, β-lactam ARGs in Antarctic and Arctic water samples were usually detected with relatively low abundance [56,60,63,76,83]. However, phenotypic resistance was observed much more frequently at moderate to really high levels. In a study investigating AMR in freshwater from areas of the Fildes Peninsula subjected to varying degrees of human influence, phenotypic β-lactam resistance was observed in all samples, but levels (especially to third-generation cephalosporins) were significantly higher in zones designated as impacted (70–90% of resistant heterotrophic isolates on the total number of investigated strains) compared to those with low human intervention (5–20%) [61]. Indeed, in most cases, resistant microorganisms belonged to human-associated groups and/or were isolated from sites under anthropogenic influence or impacted from wastewater discharge [58,59,62,67,79,81,83]. This may introduce a surveying bias between phenotypic studies on isolated strains and genotypic analyses conducted on total environmental DNA and explain, in part, the discrepancy in the levels of AMR observed by the two different types of approaches. Altogether, these data indicate that β-lactams’ resistance levels in Antarctic and Arctic water samples were comparable to what was found in other minimally impacted environments, including Argentinian lakes, South African hot springs, Hong Kong marine reserves, isolated caves in the USA, and Antarctic soils [10,121,122,123,124,125]. In a recent review, ESBL (e.g., *blaCTX-M* and *blaTEM*) and carbapenemase (e.g., *blaKPC*) genes were, respectively, reported in 70.7% and 31.7% of studies covering a range of different water bodies including rivers, seawaters, and lakes from 19 different countries [126].

### 6.3. Resistance to Sulfonamides and Trimethoprim

Sulfonamides are a group of synthetic drugs, and they represent the oldest antibacterial agents introduced in clinics. They competitively inhibit the bacterial enzyme dihydropteroate synthase (DHPS) owing to their structural analogy with *p*-aminobenzoic acid, effectively blocking folic acid biosynthetic pathway [127]. Resistance to sulfonamides is usually plasmid-mediated and linked to a non-allelic drug-resistant variant of DHPS. Three genes coding for resistant DHPS are currently known, *sul1* (usually found in class 1 integrons in association with other ARGs), *sul2* (usually found on small plasmid from the *incQ* incompatibility group), and *sul3*. In some cases, mutations leading to resistance in the chromosomally encoded DHPS gene *folP* were also reported [128]. Nowadays, sulfonamides are often combined with trimethoprim for therapeutic approaches. Trimethoprim is a synthetic molecule from the diaminopyrimidine group, which, as a structural analogue to folic acid, competitively binds to dihydrofolate reductase (DHFR), inhibiting an essential enzymatic reaction for cell metabolism (e.g., necessary for DNA thymine synthesis). As for sulfonamides, resistance to trimethoprim is also widespread and usually plasmid-mediated. More than 20 progressively numbered *dfr* genes coding for resistant variants of DHFR are currently known [128]. Sulfonamide and trimethoprim resistance was investigated in 82% and 51% of the papers examined in this review and positively detected in 56% and 26% of them, respectively (Table 3 and Figure 2), by either genotypic or phenotypic approaches. As both sulfonamides and trimethoprim are synthetic antibacterial molecules, resistance to this kind of drugs is mainly used as a proxy of human influence in pristine environments. Consistently, in most cases, it was identified in areas impacted by wastewater discharge [77,83], where related antibiotic residues were also detected [69,74], or in strains which are associated with humans’ presence (e.g., in *E. coli* strains or bacteria harboring class 1 integrons, a marker of anthropogenic impact) [21,59,65,67]. The combination of sulfonamides and trimethoprim did appear to be effective in lowering the observed phenotypic resistance levels amongst both local and human-associated bacteria [52,58]. On the other hand, *sul2* was remarkably identified in a 1200–1400-year-old Antarctic ice core sample as a part of a gene cluster including two aminoglycoside ARGs [13], and *sul1* was found ubiquitous in the Arctic Ocean [73]. Therefore, it cannot be ruled out that low levels of *sul* genes naturally occur in the background resistome of polar regions, not always solely related to human contamination. It is worth noting that sulfonamide genes were also predominant in surface sediments from the Bering Sea and Polar Research Institute [10,129] and across 21 Swiss lakes, where no significant difference in their abundance was detected between human-impacted and non-impacted areas [10,130]. Similar to tetracycline, resistance to sulfonamides was commonly reported in wastewater (especially from animal husbandry) and its various recipient systems [90,115], groundwater [38], lakes and rivers (and their sediments because of their extensive use in aquaculture) [26,37,116], estuarine and coastal environments [117], as well as in farms and soils [114].

### 6.4. Resistance to Quinolones

Quinolones are a widely used class of synthetic antimicrobials, which inhibit DNA replication by targeting two essential bacterial type II topoisomerase enzymes, DNA gyrase and DNA topoisomerase IV, which are involved in DNA supercoiling, strand-cutting and ligation [131]. Quinolone resistance can be caused by single-amino-acid mutations in the chromosomal genes coding for the target enzymes or by altered drug permeation into the cells. In other cases, quinolone resistance is mediated by proteins which mimic the DNA structure and compete for binding to the target enzymes, effectively protecting them. The *qnr* genes coding for these proteins are usually located on plasmids [132,133]. Quinolone resistance was investigated in 77% of the papers covered by this review and positively detected in 62% of them (Table 3 and Figure 2) by either genotypic or phenotypic approaches. Overall, reports about significant quinolone resistance in the Antarctic and Arctic regions appear to be frequent, with a comparable or even higher incidence than that observed worldwide, especially considering aquatic environments [57,68]. Indeed, *qnrS* was reported as one of the most abundant ARGs in Arctic tundra wetlands [77,83], while *abaQ* (encoding for a transporter involved in fluroquinolones extrusion in *Acinetobacter baumannii*) was amongst the top 50 ARG subtypes detected across freshwater and seawater samples from the Antarctic Fildes region, reaching the highest levels in seawater from the Great Wall Cove [72]. Fluoroquinolone ARGs from Antarctic lakes represented 3% of all the ARGs from that class detected across various ecosystems [55]. At a global level, quinolones and their ARGs were identified in municipal, hospital, and industrial wastewater (with higher levels reported in hospital effluents compared to municipal ones) [90,115,134]. Furthermore, it was shown that they tend to persist in aquatic environments [38,116,117], presenting a high risk of bioaccumulation. Moxifloxacin was one of the few antibiotics for which MICs above the ECOFF value (>1 mg/L) were reported amongst *Enterococcus faecalis* strains isolated from two Arctic lakes in West Spitsbergen [78]. No significant variations were observed in the levels of nalidixic acid resistance (ca. 30%) across human-impacted and non-impacted areas from King George Island, whereas for ciprofloxacin, the percentage of resistant CHB increased from 10% to 30–35%, going from zones with low degree of animal and human influences to more impacted ones [59]. Phenotypic resistance to nalidixic acid was generally more frequent and widespread than to other quinolones [52,67,79]. Therefore, microbial resistance to clinically used fluoroquinolone antibiotics in Arctic and Antarctic aquatic environments appears consistent, though less widespread than that to tetracyclines, β-lactams, and sulfonamides.

### 6.5. Resistance to Aminoglycosides

Aminoglycosides are broad-spectrum bactericidal antibiotics synthetized by *Actinobacteria*. They were amongst the first antibiotics to be discovered and used in clinics, but they are preferentially used today for topic applications due to their side effects when systematically administered. They are, however, still applied systemically in treating acute serious urinary infections, recurring respiratory infections in cystic fibrosis, and multidrug-resistant tuberculosis. They inhibit bacterial protein synthesis, interacting with 16S rRNA in the 30S ribosomal subunit and causing misreading and/or truncated proteins synthesis and cell death [135]. The main mechanism of aminoglycoside resistance involves aminoglycoside-modifying enzymes (AMEs), namely, acetyltransferases (*aac* genes), phosphotransferases (*aph* genes), and nucleotidyltransferases (*ant* genes). In addition to AMEs, other forms of resistance to aminoglycosides were described, including mutations in the 16S rRNA gene, decreased influx and/or increased efflux, and modification of the ribosomal target through methylation of 16S rRNA by methyltransferases [113,119,136]. Aminoglycoside resistance was investigated in 69% of the papers analyzed in this review and positively detected in 56% of them (Table 3 and Figure 2) by either genotypic or phenotypic approaches. As a matter of fact, aminoglycosides’ presence was not frequently investigated in aqueous environmental matrices worldwide despite their typical hydrophilic nature [37]. Nevertheless, aminoglycoside resistance was previously largely described in soils [114,125], and few reports evaluated its presence in hospital wastewater [35,115], various freshwater bodies [28] and in disinfected drinking water [35]. In the studies from our review, aminoglycoside ARGs were often detected in integron 1 gene cassettes, indicating a high mobilization risk [21,65]. Aminoglycoside ARGs of both clinical (e.g., *aacC*, *aac(3)*, *aadA*) and agricultural origin (e.g., *strA*) were also commonly detected in ice and snow samples from Arctic and Greenlandic glaciers [86]. In general, the pattern of phenotypic aminoglycoside resistance showed variations depending on the specific molecules tested [52,58,59,67,79,81]. For instance, resistance to aminoglycosides (especially to streptomycin and gentamicin) was high (>55–60%) amongst microorganisms isolated from freshwater collected in areas of the Fildes Peninsula with a significant presence of animals and humans, while much lower levels were detected in control sites. AME genes’ (*aac* and *aph*) presence was confirmed in the isolates [61]. Considering what emerged from this analysis, aminoglycoside resistance levels in the polar regions’ water samples appear to be significant, implying that this antibiotic class merits further attention and careful monitoring in Antarctica and in the Arctic, as well as worldwide.

### 6.6. Resistance to Macrolides

Macrolide antibiotics show bacteriostatic activity against Gram-positive bacteria, some Gram-negative ones, *Mycoplasma*, *Chlamydia*, *Legionella*, and *Coxiella*. As prokaryotic protein synthesis inhibitors, they bind to the ribosomal nascent peptide exit tunnel, interacting with 23S rRNA in the 50S ribosomal subunit and causing truncated proteins synthesis [137]. Several mechanisms of macrolide resistance have been described, including 23S rRNA and ribosomal proteins mutations, efflux systems (encoded by *mef*, *msr*/*mel*, and *lsa* genes) and 23S rRNA methylation by methyltransferases (*erm* genes). Macrolides can also be modified and inactivated by phosphotransferases (*mph* genes) and esterases (*ere* genes). Finally, proteins belonging to the ATP-binding cassette F (ABC-F) family were recently described, which can provide drug resistance by binding to the ribosome–antibiotic complex in the peptidyl transferase center, leading to the release of the bound macrolide and rescue of the ribosome [113,133,138]. Macrolide resistance was investigated in 69% of the papers on AMR in Antarctic and Arctic water samples and positively detected in 46% of them (Table 3 and Figure 2) by either genotypic or phenotypic approaches. Macrolides have recently been included on a watch list of substances that could pose a significant ecological risk for aquatic environments in countries within the European Union [116]. Both macrolides and macrolide resistance determinants have been frequently detected in wastewater (as they are not metabolized in the body and are mainly excreted though bile and feces as unchanged compounds) [90], estuarine and coastal environments [117], as well as various freshwater bodies [26,38,116] worldwide. This highlights the importance of investigating macrolide resistance in the supposedly pristine Antarctic and Arctic aquatic environments as well. Several macrolide ARGs (e.g., *ermC*, *ermM*) were detected in ice and snow samples from Arctic glaciers, while *mefA*/*E* was the only ARG identified in one Antarctic snow sample from Patriot Hill, a site where scientific expeditions have taken place since 1986 [86]. *ermB* (together with *sul2*) was also identified amongst microorganisms from micro-plastics-associated biofilms from an Arctic freshwater lake, but levels were higher in the surrounding water than on the microplastic substrates, suggesting it could be part of the natural background resistome of the lake [75]. Indeed, CHB associated with a polystyrene plastic macro-block deployed near the coast of Maxwell Bay showed full resistance to erythromycin and lincomycin [62]. Erythromycin also showed a MIC above the ECOFF value (>4 mg/L) amongst *E. faecalis* (14.3%) and *Enterococcus faecium* (22.2%) strains isolated from the effluent of the Polish Polar Station WWTP in West Spitsbergen [78]. The presence of macrolides (i.e., erythromycin, clarithromycin, and azithromycin) was revealed through LC-MS/MS in treated wastewater both in the Arctic [74] and in Antarctica [59], but in the relative studies, resistance to these drugs was not investigated in parallel. Therefore, an integrated and more comprehensive monitoring of their presence in Arctic and Antarctic regions and its correlation with either genotypic or phenotypic resistance appears to be needed.

### 6.7. Resistance to Chloramphenicol

Chloramphenicol was first isolated from *Streptomyces venezuelae* in 1947, but due to its relatively simple structure, it has been marketed as a product of chemical synthesis ever since (together with its fluorinated derivative florfenicol). It is a broad-spectrum bacterio-static antibiotic but its use in humans has been replaced by other antibiotics because of its severe adverse effects. Chloramphenicol is a protein synthesis inhibitor, preventing protein chain elongation by reversible binding to the peptidyl transferase center at the 50S ribosomal subunit of 70S ribosomes [139]. The first and most common mechanism of resistance to chloramphenicol is its enzymatic inactivation by acetylation, performed by chloramphenicol acetyltransferases (CATs, encoded by *cat* genes). Resistance to chloramphenicol through efflux systems is also frequent and can be mediated by both specific transporters (e.g., *cml*, *cmr*, *floR* genes) and multidrug ones. Finally, inactivation by phosphotransferases and mutations in 23S rRNA or ribosomal proteins, though rarer, have also been reported [140]. Chloramphenicol resistance was investigated in 69% of the papers covered in this review and positively detected in 33% of them (Table 3 and Figure 2). Chloramphenicol resistance was almost never described in Arctic and Antarctic aquatic environments through genotypic approaches, but mainly following isolation procedures and phenotypic screening. Chloramphenicol ARGs were unfrequently identified in a Greenlandic glacier [86], wastewater from WWTPs in Canadian Arctic [76], and freshwater and seawater samples from the Antarctic Fildes Peninsula [72]. Phenotypic resistance to chloramphenicol was completely absent amongst Antarctic wastewater- and seawater-isolated *E. coli* strains [58,59,67], as well as amongst CHB and coliforms from seawater in Kongsfjorden [81]. On the other hand, relatively high chloramphenicol resistance was reported amongst CHB from the Pasvik River [74], from the Antarctic Sea (67%) [62], and from freshwater samples subjected to a significant degree of human and animal influence in King George Island (85% incidence of resistant strains, though resistance amongst isolates from unimpacted freshwater samples was only 5%) [61]. Worldwide, the presence of chloramphenicol-resistant bacteria and ARGs was also reported in other aquatic environments, including wastewater secondary reception systems [115], rivers and lakes [28], though more rarely than for other groups of antibiotics [116]. This was unexpected considering the fact that some common chloramphenicol ARGs, such as *floR*, *catII*, *catB9*, and *catB2*, actually originated in aquatic bacterial genera, such as *Photobacterium*, *Vibrio*, and *Shewanella* [141].

### 6.8. Resistance to Glycopeptides

Glycopeptides are glycosylated non-ribosomal heptapeptides, frequently used as last-resort drugs to treat life-threatening infections caused by multidrug-resistant Gram-positive pathogens, such as *S. aureus*, *Enterococcus* spp., and *Clostridiodes difficile*. Glycopeptides are bacterial cell wall synthesis inhibitors, which bind to the d-Alanine-d-Alanine (d-Ala-d-Ala) dipeptide terminus of the peptidoglycan precursors, blocking the subsequent transpeptidation and transglycosylation reactions required for peptidoglycan cross-linking [142]. While Gram-negative bacteria are intrinsically resistant to glycopeptides because they are impermeable to them, the main mechanism of resistance in Gram-positive bacteria involves remodeling of the terminal d-Ala-d-Ala to either d-Alanine-d-Lactate (d-Ala-d-Lac) or d-Alanine-d-Serine (d-Ala-d-Ser). The first substitution produces a 1000-fold reduction in the affinity of glycopeptides for their molecular target and is mediated by *vanHAX* genes (together with accessory genes *vanZ* and *vanY*, and *vanRS*, a two-component regulatory system). The second substitution, in contrast, confers only moderate resistance as it causes a 6-fold reduction in affinity and is mediated by the *vanT*, *vanC*, and *vanXY* genes. Intermediate resistance has also been linked to mutations leading to a thickened cell wall and low permeability in staphylococci [133,136,142,143]. Glycopeptide resistance was investigated in 59% of the papers on AMR in Antarctic and Arctic water samples and positively detected in 33% of them (Table 3 and Figure 2) by either genotypic or phenotypic approaches. Vancomycin-related ARGs are relatively common amongst soil-dwelling microorganisms producing glycopeptides [144,145] but were also identified in a 30,000-year-old frozen sediment core [146]. Moreover, they are frequently carried by *Enterococcus* species, which represent a growing concern because of their widespread presence in clinical settings [142,143], hospital and community wastewater [134], as well as in rivers [28]. Resistance to glycopeptides does not appear common in polar aquatic environments, and a study investigating AMR in *Enterococcus* species isolated from West Spitsbergen (Arctic) did not report MIC above the ECOFF value for vancomycin [78]. Glycopeptide ARGs were rarely identified in Canadian Arctic tundra wetlands [76,77,80] and in Arctic and Antarctic glaciers [27,68,70,86]. However, they were found enrichened in Arctic seawater [71] and in one Antarctic lake, which was the source of 12% of all the glycopeptide ARGs identified across several agricultural and non-agricultural metagenomes (a significantly higher value than reported for any other antibiotic class) [56]. Moreover, it is also true that phenotypic resistance to glycopeptides was not routinely tested in studies investigating antibiotic resistance in the Arctic and in Antarctica, or amongst human-associated bacteria. Nevertheless, strains isolated from polystyrene in King George Island were fully resistant to both vancomycin and teicoplanin [62], and the same was true for isolates from polyvinyl-chloride and polyethylene in Terra Nova Bay, while bacteria obtained from seawater at the same sites showed full sensitivity to glycopeptide antibiotics [54]. So, while resistance to glycopeptides in the polar regions does not appear concerning as of yet, increased anthropogenic activities and pollution (e.g., microplastics) may favor the acquisition and spread of the genetic determinants naturally present amongst local microbial populations, increasing the risk of transmission to pathogenic bacteria and for human health.

### 6.9. Resistance to Rifamycins

Rifamycins were first described in the 1950s as secondary metabolites produced by the soil actinomycete *Amycolatopsis rifamycinica*. They display broad-spectrum activity against mycobacteria and Gram-positive bacteria. Rifamycin semisynthetic derivatives (i.e., rifampicin, rifabutin, and rifapentine) are included in the World Health Organization’s (WHO) Essential Medicines as first-line antibiotics for the treatment of tuberculosis. They inhibit bacterial transcription by targeting RNA polymerase, binding to its β subunit (encoded by *rpoB* gene) in the DNA/RNA exit tunnel and sterically blocking mRNA chain extension [147]. In pathogenic bacteria, resistance to rifamycins usually arises through *rpoB* mutations, which decrease the binding affinity of the antibiotic, while in environmental bacteria, enzyme inactivation of rifamycins catalyzed by ADP ribosyltransferases (*arr* genes), glycosyltransferases (*rgt* genes), phosphotransferases (*rph* genes), and monooxygenases (*iri* and *rox* genes) has also been observed [148]. Rifamycin resistance was investigated in 49% of the papers examined in this review and positively detected in 26% of them (Table 3 and Figure 2) by either genotypic or phenotypic approaches. In general, rifamycin resistance does not appear widespread in pristine or aquatic environments or in natural bacterial communities, but it still represents a growing concern because of its common association in clinical settings with pathogenic mycobacteria, such as *Mycobacterium tuberculosis* and *Mycobacterium leprae* [149,150]. In the polar regions’ aquatic environments, *rpoB* mutants were found amongst the most abundant ARG subtypes in Kongsfjorden seawater sediments [84], King George Island seawater and freshwater samples [72], and in the Arctic Ocean [28], whereas phenotypic resistance to rifampicin was only detected in 10–15% of isolates from Pasvik River [79] and Thala Hills ponds [52].

### 6.10. Resistance to Other Antibiotics

Other antibiotics, mostly belonging to classes comprising just a single or few members, were investigated in less than half of the papers covered in this review, through either genotypic or phenotypic approaches (Table 3 and Figure 2). Many of these are actually older drugs, which are nowadays being rediscovered and/or repurposed as a result of the rampant threat of multidrug-resistant pathogens and the lack of novel antibiotic candidates on the market. For example, fosfomycin, which was previously used mainly to treat uncomplicated urinary infections, is attracting clinicians’ attention worldwide [151], while nitrofurans, owing to their multi-activity under aerobic and anaerobic conditions, are being repurposed from agricultural chemicals and basic antibiotics to efficient therapies against human-life-threatening diseases [152]. Concerningly enough, resistance to fosfomycin and nitrofurantoin (when investigated) was identified in 23% and 13% of the papers analyzed in this review (Table 3 and Figure 2), with high levels of phenotypic resistance (70–100% of screened isolates) identified in both Antarctic and Arctic waters [54,62,79]. Nitrofurantoin had MIC above ECOFF values amongst Arctic *Enterococcus* species [78]. Similar results were observed for linezolid, a member of oxazolidinones, the only new class of synthetic antibiotics advanced in clinical use over the past 50 years [153], against which resistance was identified in 5% of the papers (Table 3 and Figure 2) [54,62]. Resistance to polymyxins, a class of peptide antibiotics recently repurposed as last-line therapeutic options against Gram-negative resistant bacteria [154], was detected in 13% of the papers (Table 3 and Figure 2), but the described levels generally appeared to be low [54,68,79,81,83] in spite of the rising number of polymyxin-resistant strains reported worldwide [154]. Overall, it appears necessary to expand and further integrate the range of antibiotics for which resistance is routinely searched in studies investigating AMR in the Antarctic and Arctic environment to include even less common ones. Indeed, understanding whether local communities already harbor resistance determinants against last-resort and/or rarely prescribed drugs may help in anticipating future scenarios about AMR evolution and how to face them.

### 6.11. Multidrug Efflux Systems, Integrons, and HGT

Finally, multidrug efflux systems can confer resistance to several antibiotic classes by reducing the drug intracellular concentration and allowing bacteria to tolerate higher extracellular concentration of it. They are especially relevant in Gram-negative bacteria and can either act independently or add their effect to the more specific mechanisms of AMR described above. Efflux pumps can be divided into two groups: primary transporters such as ATP-Binding Cassette (ABC) family transporters (e.g., MacB, SmdAB, AbcA), which use the energy of ATP binding and hydrolysis for efflux, or secondary transporters, such as the Major Facilitator Superfamily (MFS, e.g., CraA, MdfA, EmrB), Small Multidrug Resistance (SMR, e.g., AbeS) family, Resistance Nodulation Division (RND, e.g., AcrAB-TolC, MexAB-OprM, CmeABC, MtrCDE) family and Multidrug and Toxic Compound Extrusion (MATE, e.g., NorM-VC, VcmN) family, which use the electrochemical potential of the membrane (either sodium ions or protons gradients) for efflux [155]. Multidrug efflux systems were investigated in 41% of the papers on AMR in Antarctic and Arctic water samples and positively detected in 33% of them (Table 3 and Figure 2). In particular, multidrug-efflux-related ARGs were identified in the Antarctic sea [55,57,60,71,72] and in Antarctic lakes [56,71,72], as well as in the Arctic sea [28,84] and in Arctic lakes [63,76,80], often representing the most abundant ARG subtype detected [28,57,68,72,80,84] and positively correlating with the *intI1* gene [60]. Indeed, as previously mentioned, integrons are considered a marker for both AMR, HGT, and anthropogenic impact. They were investigated in 54% and identified through genotypic approaches in 23% of the studies covered in this review, respectively (Table 3 and Figure 2). When detected, they were found to be in close association with ARGs, especially belonging to sulfonamide, aminoglycoside, and tetracycline classes [21,56,60,65,69,77,80,83], highlighting their significant HGT potential in these environments. From this point of view, several studies employing genotypic approaches for AMR monitoring tried to further evaluate whether the detected ARGs possessed a high transmission risk as well. Of particular interest was the analysis of their location and genetic context; for instance, whether they were placed near MGEs, on plasmids, or in the genome from viral particles, which would favor their exchange between different hosts through HGT mechanisms such as transformation and transduction. Indeed, Cuadrat et al. (2020) found that 25% of the putative ARGs they identified from oceanic water samples were present in contigs classified as plasmids. Moreover, a significant proportion of these contigs contained two or more (up to five) different ARGs at the same time, suggesting the presence of multi-resistant microorganisms in these environments [55]. Similarly, Makowska-Zawierucha et al. (2024), who focused their attention precisely on the plasmidome associated with glacial ice and adjacent aquatic environments across the high Arctic archipelago of Svalbard, reported that ARGs against the main antibiotic classes were present in the recovered plasmids. The number and diversity of these ARGs increased in response to anthropogenic inputs, and they often correlated with MRGs, virulence genes, and integrons as well [80]. Likewise, studies investigating viral ecology in polar habitats consistently identified ARG sequences in viral genomes [27,28,53]. For instance, ARG-containing phage particles were more prevalent among Antarctic seawater samples than Mediterranean ones and Antarctic bacterial communities were confirmed as their source [53]. Active transmission of ARGs between viral and bacterial hosts from Arctic glacier was demonstrated as well, with increased solar radiation and emission of carbon dioxide influencing the release of ARGs and viruses towards downstream habitats [27]. Thus, the results from these studies emphasize the importance of tracking not only ARG presence but their sources and dissemination routes as well.

## 7. Conclusions

The supposedly pristine polar regions, with their extreme environmental conditions, have been consistently receiving more attention from the scientific community for ecological reasons and for the dramatic impact of climate change on them. Due to the ongoing rapid changes in polar ecosystems, it is consequently important to assess the current composition of local microbial communities, and, in particular, the spread among them of AMR, which represents an increasingly alarming matter worldwide [156]. The analysis of AMR in these remote regions, which still show low anthropogenic influence, may help to clarify the evolution of this phenomenon, and, at the same time, lead to a better understanding of the origins of ARGs beyond clinical conditions. Indeed, the presence of ARGs in these areas of the world cannot be solely attributed to human influence, as AMR is firstly a natural defense mechanism of bacteria [27,28,54,57,68], and ARGs have also been identified in samples predating the antibiotic era [13,146]. However, AMR was found more dominant in polar areas where human and wildlife activities have been consistently reported (e.g., tourism, research stations, old sewerage systems, microplastics, animal migration) compared to more pristine ones [61,156]. In this regard, the aquatic environment plays a particularly important role as it can collect ARGs released via WWTPs, and then act as a reservoir and/or actively participate in their dissemination [117]. This phenomenon seems especially relevant in the polar regions where wastewater disposal practices are antiquated.

**Figure 3:**
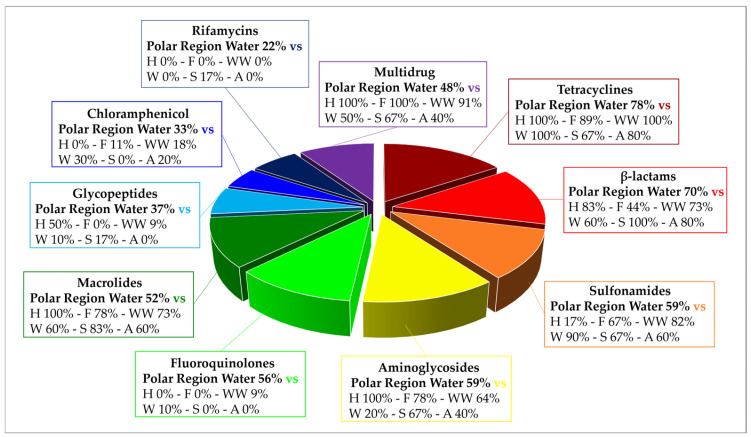
Frequency of detection of antibiotic resistance genes (ARGs) against the main antibiotic classes through genotypic approaches (i.e., PCR, qPCR, metagenomics) in Arctic and Antarctic water samples in the AMR studies covered in this review, expressed as number of genotypic studies in which the antibiotic class is detected per total number of analyzed genotypic studies (27). The AMR values calculated in this review are compared to those reported in the review by Zhuang et al. (2021) [114], focused on AMR across the world in different types of environments: human feces (H), farms (F), wastewater treatment plants (WW), water (W), soil (S), and air (A). The values were extrapolated from 47 papers manually analyzed by the authors [114], covering qPCR and metagenomic AMR studies from five regions: Asia, Europe, the United States of America, Africa, and Australia.

**Figure 4:**
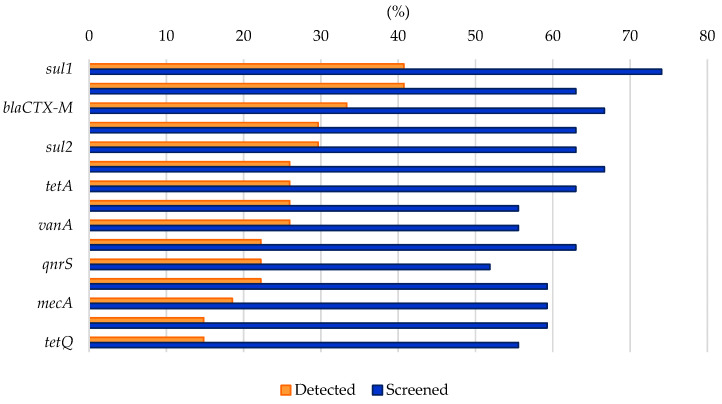
Frequency of screening and detection of the top 15 ARGs identified in the studies investigating AMR in Arctic and Antarctic water samples through genotypic approaches (i.e., PCR, qPCR, metagenomics) covered in this review, expressed as number of genotypic studies in which the ARG is screened/detected per total number of analyzed genotypic studies (27).

In general, the families of antibiotics against which resistance was most commonly detected in Antarctic and Arctic waters were the same traced around the globe [114]. Tetracycline, β-lactam, and sulfonamide resistance was predominant, as well as macrolide and efflux-system-mediated multidrug resistance (Figure 3) [114]. Moreover, aminoglycoside and fluroquinolone resistance appeared to have a higher incidence in the polar regions aquatic environment compared to that from other areas of the world (which was true to a lesser degree for glycopeptide and rifamycin antibiotics as well) (Figure 3) [114]. Oftentimes, the number of different ARG subtypes detected in these supposedly pristine environments was comparable to that from more human-impacted areas [84]. Accordingly, the top 15 ARGs reported in the aquatic environment from the North and South Pole mainly belonged to the same classes as those identified in Asia, Europe, the USA, Africa, and Australia [114], namely, sulfonamides (*sul1*, *sul2*), β-lactams (*blaTEM*, *blaCTX-M*, *mecA*), tetracyclines (*tetO*, *tetA*, *tetB*, *tetD*, *tetQ*), aminoglycosides (*aadA*), and glycopeptides (*vanA*) (Figure 4). As a matter of fact, a recent global survey using high-throughput qPCR to map richness and proportion of ARGs in the soil, one of the most important ARGs reservoirs on Earth, revealed how Antarctic soils and extreme cold environments possessed a particularly high proportion of ARGs, between 2 and 9 times higher than that of other ecosystems [157]. This could possibly be due to co-evolution mechanisms aimed at providing bacteria with ways to face antibiotic- and cold-related stressors at the same time. Similar factors might have contributed to the high levels of resistance detected in Antarctic and Arctic waters as well.

Nevertheless, it has to be considered that some of the genes that are identified as ARGs in AMR studies may actually play other roles in the natural environment, for example, being involved in biogeochemical cycles, or their function may actually depend on the context in which they are placed and whether there are other genes involved in AMR nearby [157,158]. Therefore, the detection of a specific ARG in a given environment, even in regions which are considered mostly untouched by anthropogenic influence, does not immediately signify that it will represent a risk for human health. Only a small percentage of ARGs from all kinds of environments were recently classified as “risk ARGs” based on strict human accessibility, mobility, human pathogenicity, and clinical availability criteria [158]. On the other hand, ARG dissemination across habitats and transfer from non-pathogenic bacteria to pathogens consistently increased with the intensity of anthropogenic activities and positively correlated with the abundance of MGEs [148], such as the *intI1* gene, as was highlighted in this review as well [21,56,65,69,77,84]. The role of non-pathogenic environmental bacteria as a silent reservoir for ARGs, and the existence of environmental co-resistomes have indeed been reported from other extreme ecological niches as well, from hot springs to sea brines [159,160]. As anthropic activities in polar regions have intensified recently and ARG presence has often been reported in association with MGEs, the importance of monitoring these environments cannot be underestimated. Our hope is that this review may help convey this message, describing the different advantages of the various approaches available to monitor AMR in polar water environments, promoting further and more extensive studies about it, and contributing to drawing a global picture of AMR.

## 8. Research Future Directions

From this review, it emerges that several steps still need to be taken to reach the goal of a more complete surveillance of AMR in the polar regions, allowing to monitor the situation and promptly devise strategies to mitigate and control it when necessary. Firstly, the number of studies focusing on characterizing AMR in the polar regions across different types of samples, from terrestrial to marine and wildlife-associated ones, needs to be expanded, systematically exploring both pristine and under anthropogenic influence areas. Indeed, there is still a significant lack of understanding regarding which factors actually influence the persistence and transmission of ARGs, especially in these extreme environments, and how they may interact with each other. A more consistent investigation into the presence of antibiotic molecules and other pollutants in the polar regions could also help in evaluating AMR distribution and xenobiotics effects on local microbial communities. It is clear that the untreated or partially treated wastewater released from research stations and other human settlements in Antarctica and in the Arctic has a significant impact on its receiving environment. A survey from the Swedish Polar Research Secretariat showed that 37% of permanent research stations and 69% of summer ones operating in Antarctic lacked any form of treatment facility [161]. Current guidelines should therefore be implemented to set higher threshold values for released water quality and more efficient wastewater treatment systems should be required. For instance, physiochemical treatments, in addition to biological ones, and dry flush toilets have recently been proven as feasible alternatives to facilitate co-treatment of wastewater along with other organic waste fractions and provide environmentally safe and easy-to-handle by-products [162,163]. Routine qPCR controls could also be conducted to monitor wastewater and downstream environments in a quick and cost-effective way by selecting appropriate ARGs as biomarkers [164]. Research stations, where NGS instruments are already available for research purposes, could implement regular metagenomics controls on wastewater ARGs content as well [165]. Overall, as outlined by the SCAR Codes of Conduct for field research, while the advancement of scientific research in these regions must be ensured, protocols must be devised to guarantee that scientists and other people engaged in Arctic and Antarctic expeditions have as little environmental impact as possible, safeguarding polar habitats for future generations [166]. In this context, both research and recreational activities should be carried out responsibly to avoid exacerbating the role of human activities in ARB and ARGs dissemination.

Secondly, analytical procedures need to be standardized to make it possible to integrate and compare data from different studies. From our analysis, it appears that combining genotypic and phenotypic approaches may be the wisest choice to obtain a truly comprehensive overview of the AMR, exploiting to the maximum the strengths of the different techniques and compensating for their limitations. On this basis, standard protocols (e.g., similar to those provided by CLSI for the Kirby–Bauer assay) should be developed and shared online, covering all phases of an AMR study, from how to collect, store, and process environmental samples to which techniques and conditions to select to analyze them and interpret their results [167]. This seems, in particular, necessary for genotypic approaches, where the applied protocols and corresponding bioinformatic pipelines present a much higher variability than those for phenotypic ones, making them harder to compare. Guidelines such as those offered by the WHO Global Antimicrobial Resistance and Use Surveillance System (GLASS), launched to provide a standardized approach to the collection, analysis, interpretation and sharing of data by countries involved in AMR surveillance in human and clinical environments, could be taken as reference and expanded to be applied to the polar regions as well [168].

Innovative techniques such as mass-spectrometry-based methods, microfluidics, dPCR, and functional metagenomics have been gaining attention in recent years because of the many possibilities they offer in terms of large-scale identification of ARB, rapid analysis of huge datasets, and discovery of novel ARG categories [169,170]. Artificial intelligence and machine learning also represent new ways to address the challenges posed by AMR, for instance, by predicting resistance patterns, rapidly integrating data from multiple sources to map the global distribution of AMR and support researchers in understanding how ARB and ARGs geographically spread [171]. While there are no examples of their application to the polar regions’ environment as of yet, they will certainly play a central role in the future in AMR monitoring, even in these most remote areas. The bias we remarked on towards the more frequent isolation and testing of human-associated microorganisms rather than autochthonous ones should be addressed as well. Finally, as more and more data are gathered and published, a global dedicated network should be created to report and contextualize them, allowing to determine and predict ARGs and AMR trends, not just at a local level, but globally and over time.

In conclusion, Antarctica and the Arctic inclusion in AMR surveillance programs appears to be of the utmost importance to build a vaster and truly comprehensive portrait of this global health problem and promote more incisive and timely decisions to try to stem and fight against AMR before it is too late.

## Figures and Tables

**Table 1:** Comparison between the different techniques available to study AMR in the polar regions in terms of sensibility, specificity, coverage, cost, and applicability. A discrete scale was used to categorize the different approaches, with “+” indicating a low level, “++” intermediate, “+++” high, and “++++” very high.

Technique	Sensibility	Specificity	Coverage	Cost	Applicability
Genotypic approaches					
PCR	+++	+++	++	+	Yes
qPCR	++++	++++	+++	++	Yes
dPCR	++++	++++	+++	++	Yes
Whole-genome sequencing	++++	+++	++++	+++	Yes
Shotgun sequencing	++	+++	++++	++++	Yes
Phenotypic approaches					
Disk diffusion test	+++	++	++	++	Yes
MIC	++++	++++	++	+++	Yes

**Table 2:** General overview of the antimicrobial resistance (AMR) studies covered in this review, investigating water samples from polar regions. All papers—listed in alphabetical order—were retrieved from Pubmed, using the query “antibiotic resistance + polar/Arctic/Antarctic + water/marine/sea/seawater/ocean/lake/pond/wastewater/ice” and have been published in the timeframe from January 2010 to January 2025. Details about geographical sampling sites, type of samples, and selected approaches for AMR screening are herein reported.

Paper	Geographical Location	Type of Sample (Water)	Type of Sample (Other)	Culture-Dependent Isolation	Screening Approach	Reference
Akulava et al. (2022)	Antarctica (Vecherny Region, Thala Hills Oasis, Enderby Land)	Ice and Snow	-	Yes	Phenotypic (disk diffusion test)	[52]
Blanco-Picazo et al. (2020)	Antarctica (Livingston Island, South Shetland Islands)	Seawater	Animals (fishes and shellfishes)	No	Genotypic (qPCR)	[53]
Bonanno Ferraro et al. (2024)	Arctic (Svalbard Archipelago)	Seawater	-	No	Genotypic (dPCR)	[73]
Cao et al. (2020)	Arctic and Antarctica	Seawater	-	No	Genotypic (NGS metagenomic sequencing)	[85]
Caruso et al. (2024)	Antarctica (Ross Sea, Terra Nova Bay)	Seawater (Plastisphere)	-	Yes	Phenotypic (disk diffusion test)	[54]
Chaves-Barquero et al. (2016)	Arctic (Cambridge Bay, Nunavut, Canadian Arctic)	Wastewater and Seawater	-	No	Genotypic (qPCR)	[74]
Cuadrat et al. (2020)	Antarctica	Seawater	-	No	Genotypic (archived metagenomic data bioinformatic analysis)	[55]
Durso et al. (2012)	Antarctica	Freshwater	-	No	Genotypic (archived metagenomic data bioinformatic analysis)	[56]
Giovannini et al. (2024)	Antarctica (Ross Sea, Terra Nova Bay)	Seawater	-	Yes	Genotypic (NGS metagenomic sequencing)	[57]
González-Pleiter et al. (2021)	Arctic (Ny-Ålesund, Kongsfjorden, Spitsbergen, Svalbard Archipelago)	Freshwater (Plastisphere)	Sediments	No	Genotypic (qPCR)	[75]
Gromala et al. (2021)	Arctic (Baker Lake, Cambridge Bay and Kungluktuk, Nunavut, Canadian Arctic)	Wastewater and Freshwater	-	No	Genotypic (NGS metagenomic sequencing)	[76]
Hayward et al. (2018)	Arctic (Sanikiluaq and Naujaat, Nunavut, Canadian Arctic)	Wastewater and Seawater	Soil	No	Genotypic (qPCR)	[77]
Hernández et al. (2012)	Antarctica (Bernardo O’Higgins Station on the Antarctica Peninsula, Arturo Prat Station on Greenwich Island and Fildes Bay on King George Island, South Shetland Islands)	Seawater	Animals (penguin feces)	Yes	Combined (qPCR and disk diffusion test)	[58]
Hernández et al. (2019)	Antarctica (King George Island, South Shetland Islands)	Wastewater and Seawater	-	Yes	Phenotypic (disk diffusion test)	[59]
Jang et al. (2022)	Antarctica (Ross Sea, Terra Nova Bay)	Seawater	-	No	Genotypic (qPCR)	[60]
Jara et al. (2020)	Antarctica (Fildes Peninsula, King George Island, South Shetland Islands)	Freshwater	-	Yes	Combined (PCR and disk diffusion test)	[61]
Kalinowska et al. (2021)	Arctic (Stanislaw Siedlecki Station, South Spitsbergen National Park, Isbjornhamna Bay, Hornsund Fjord, Svalbard Archipelago)	Wastewater and Freshwater	Sediments	Yes	Phenotypic (MIC)	[78]
Laganà et al. (2018)	Arctic (Pasvik River, Norway)	Freshwater	Sediments	Yes	Phenotypic (disk diffusion test)	[79]
Laganà et al. (2019)	Antarctica (Maxwell Bay, King George Island, South Shetland Islands)	Seawater (Plastisphere)	-	Yes	Phenotypic (disk diffusion test)	[62]
Liu et al. (2023)	Arctic (Borup Fiord Pass, Nunavut, Canadian Arctic)	Ice	Cryoconite	No	Genotypic (archived metagenomic data bioinformatic analysis)	[28]
Makowska-Zawierucha et al. (2024)	Arctic (Kongsfjord, Spitsbergen, Svalbard Archipelago)	Ice, Freshwater, and Wastewater	Sediments	No	Genotypic (NGS metagenomic sequencing)	[80]
Mohamed Hatha et al. (2015)	Arctic (Kongsfjord, Spitsbergen, Svalbard Archipelago)	Seawater	Sediments	Yes	Phenotypic (disk diffusion test)	[81]
Neethu et al. (2015)	Arctic (Kongsfjord, Spitsbergen, Svalbard Archipelago)	Seawater	Sediments	Yes	Phenotypic (disk diffusion test)	[82]
Neudorf et al. (2017)	Arctic (Pond Inlet, Clyde River, Iqaluit, Baffin Island, Nunavut, Canadian Arctic)	Wastewater and Seawater	-	No	Genotypic (qPCR)	[83]
Okubo et al. (2019)	Antarctica (Dome Fuji Station, Queen Maud Land)	Ice	-	No	Combined (PCR and MIC)	[13]
Otur et al. (2023)	Antarctica (Ardley Island, King George Island, South Shetland Islands)	Freshwater	-	Yes	Combined (NGS whole-genome sequencing and disk diffusion test)	[63]
Otur et al. (2024)	Antarctica (Ardley Island, King George Island, South Shetland Islands)	Freshwater	-	Yes	Combined (NGS whole-genome sequencing, qPCR, and disk diffusion test)	[64]
Power et al. (2016)	Antarctica (Davis Station, Vestfold Hills)	Seawater	Sediments and Animals (marine invertebrates, seals and penguin feces)	Yes	Genotypic (PCR and cassette PCR)	[65]
Presta et al. (2016)	Antarctica (Dumont D’Urville Station, Petrel Island)	Seawater	-	Yes	Genotypic (NGS whole-genome sequencing)	[66]
Rabbia et al. (2016)	Antarctica (King George Island, South Shetland Islands)	Wastewater and Seawater	Animals (migratory birds feces)	Yes	Phenotypic (disk diffusion test)	[67]
Ren et al. (2024)	Antarctica (Fildes Peninsula, King George Island, South Shetland Islands)	Snow	-	No	Genotypic (NGS metagenomic sequencing)	[68]
Segawa et al. (2013)	Arctic (Austfonna, Bowdoin, Qaanaaq, Pakitisoq, Russell from Greenland and Svalbard Archipelago) and Antarctica	Ice and Snow	-	No	Genotypic (qPCR)	[86]
Stark et al. (2016)	Antarctica (Davis Station, Vestfold Hills)	Wastewater and Seawater	Sediments and Animals (marine invertebrates, seals and penguin feces)	Yes	Genotypic (PCR and cassette PCR)	[21]
Szopińska et al. (2022)	Antarctica (King George Island, South Shetland Islands)	Wastewater and Seawater	-	No	Genotypic (qPCR)	[69]
Ushida et al. (2010)	Antarctica (Rink Crags, James Ross Island)	Ice	Cryoconite	No	Genotypic (qPCR)	[70]
Wang et al. (2024)	Arctic (Canadian Basin, Greenland, Svalbard Archipelago)	Seawater	Cryoconite	No	Genotypic (archived metagenomic data bioinformatic analysis)	[27]
Yang et al. (2019)	Antarctica (Fryxell Lake, Bonney Lake and Ross Sea)	Seawater and Freshwater	-	No	Genotypic (archived metagenomic data bioinformatic analysis)	[71]
Zhang et al. (2022)	Arctic (Kongsfjord, Spitsbergen, Svalbard Archipelago)	Seawater	Sediments	No	Genotypic (NGS metagenomic sequencing)	[84]
Zhang et al. (2022b)	Antarctica (Ardley Island, Ardley Cove, Great Wall Cove, King George Island, South Shetland Islands)	Seawater and Freshwater	-	No	Genotypic (NGS metagenomic sequencing)	[72]

Quantitative Polymerase Chain Reaction (qPCR). Next-Generation Sequencing (NGS). Digital Polymerase Chain Reaction (dPCR). Polymerase Chain Reaction (PCR). Minimum Inhibitory Concentration (MIC).

**Table 3:** List of antibiotic molecules and antibiotic resistance genes (ARGs), grouped by the main known antibiotic classes, found in the various studies on AMR in Arctic and Antarctic water samples covered in this review. The mode of action of the antibiotic class is indicated. The reference works, where the antibiotic molecules and/or the ARGs were investigated (screening) and/or detected (detection), are reported, with percentage values referring to the number of studies in the category per total number of analyzed studies (39).

Antibiotic Class	Mode of Action	Antibiotic Molecules	ARGs	References (Screening)	References (Detection)
Aminoglycosides	Protein Synthesis Inhibition	Amikacin, Gentamicin, Kanamycin, Kasugamycin, Neomycin, Netilmicin, Sisomicin, Streptomycin, Tobramycin	*aac(2′)-Ia/b/c*, *aac(2′)-IIa*, *aac(3)*, *aac(3)-IIa*, *aac(3)-IVa*, *aac(3)-VIIIa*, *aac(3)-Ib*, *aac(3)-IIb*, *aac(3)-IIIc*, *aac(6′)*, *aac(6′)-31/32*, *aac(6′)-Ia/b*, *aacC1/2/4/9*, *aadA*, *aadA1a/2/4/5/6/7/10*, *aadB*, *aadE*, *aadK*, *amrB*, *ant*, *ant(2”)-Ia*, *ant(3”)-Ia*, *ant(4′)*, *ant(4′)-IIa/b*, *aph(2′)-Ib/d*, *aph(3′)*, *aph(3′)-Ia*, *aph(3′)-IVa*, *aph(3′)-VIa*, *aph(3′)-Vb*, *aph(3”)-Ib (strA)*, *aph(4)-Ia/b*, *aph(6)*, *aph(6)-Ia*, *aph(6)-Id (strB)*, *aph(9)-Ia*, *armA*, *cpaA*, *rpsL*, *strW*, *strX*	69% [13,27,28,52,54,55,56,57,58,59,61,62,63,64,66, 67,68,70,71,72,76,79,80,81,84,85,86]	56% [13,21,27,28,52,54,56, 57,58,59,61,63,66,67,68,70,72,76,79,80,81,86]
β-lactams					
Carbapenems	Cell Wall Biosynthesis Inhibition	Ertapenem, Imipenem, Meropenem	*ampC*, *blaACC*, *blaACT-1*, *blaADC-82*, *blaAST-1*, *bla-B*, *blaBIC-1*, *blaBIL-1*, *blaBJP-1*, *blaCAM-1*, *blaCARB-5/16*, *blaCFI*, *blaCIT*, *blaCKO-1*, *blaCMH-1*, *blaCMY-1/2/3/4/5/6/7/8/9/10/11*, *blaCTX-M-1/2/8/9/25/63*, *blaDHA-1/2/16*, *blaEBC*, *blaEC*, *blaEXO-1*, *blaFOX-1/2/3/4/5B*, *blaFTU-1*, *blaGES*, *blaIMP-1*, *blaKPC-9*, *blaLAT-1/2/3/4*, *blaMIR-IT*, *blaMOX-1/2/5*, *blaMSI-1*, *blaOXA-22/29/53/158/173/240/258/279/296/328/375/724*, *blaPDC-91*, *blaPER-2*, *blaPNGM-1*, *blaRAHN*, *blaROB-1*, *blaRTG*, *blaSGM*, *blaSHV*, *blaTEM*, *blaVEB*, *mecA*, *mecD*, *mrcA*, *pAmpCDHA*, *pAmpCFOX*, *pbpC*, *pbpE*, *per-I*	87% [13,27,28,52,53,54,55,56,57,58,59,60,61,62,63,64,66,67,68,70, 71,72,73,76,77,78,79,80,81,82,83,84,85,86]	69% [27,52,53,54,55,56,57,58,59,60,61,62,63,64,67,68,71, 72,73,76,77,79,80,81,82,83,84]
Cephalosporins		Cefaclor, Cefalotin, Cefazolin, Cefepime, Cefoxitin, Cefotaxime, Cefpodoxime, Ceftazidime, Ceftriaxone, Cefuroxime			
Monobactams		Aztreonam			
Penicillins		Amdinocillin, Amoxycillin, Ampicillin, Carbenicillin, Methicillin, Mezlocillin, Oxacillin, Penicillin G, Piperacillin			
Chloramphenicol	Protein Synthesis Inhibition	Chloramphenicol, Florfenicol	*cat1/2/3/4*, *catA3/4/6/9/10/12/14/16*, *catB1/2/3/4/5/6/7/8/9*, *cfrA*, *cfrC*, *cmlA*, *cmlB*, *cml(E-5)*, *cmlV*, *cmrA*, *cmrx*, *cmx*, *floR*, *pexA*	69% [13,27,28,52,54,55,56,57,58,59,61,62,63,64,66, 67,68,70,71,72,76,79,80,81,84,85,86]	33% [27,52,54,61,62,64,66,68,71,72, 76,79,86]
Fosfomycin	Cell Wall Biosynthesis Inhibition	Fosfomycin	*fosA*, *fosB*, *murA*	46% [27,28,54,55,56,57,58,62, 63,64,66,68,71,72,76,79,80,85]	23% [27,54,56,62,68,71,72,79,80]
Fluoroquinolones	DNA Replication Inhibition	Cinoxacin, Ciprofloxacin, Levofloxacin, Moxifloxacin, Nalidixic Acid, Norfloxacin, Ofloxacin, Pipemidic Acid	*abaQ*, *gyrA*, *mfd*, *parE*, *patA*, *qepA*, *qnrA*, *qnrB*, *qnrC*, *qnrD*, *qnrS*, *qnrVC4*	77% [13,27,28,52,54,55,56,57,58,59,60,61,62,63,64,66, 67,68,71,72,76,77,78,79,80,81,82,83,84,85]	62% [28,52,54,56,58,59,60,61,62,63,66, 67,68,71,72,76,77,78,79,80,81,82,83,84]
Glycopeptides	Cell Wall Biosynthesis Inhibition	Bleomycin, Teicoplanin, Vancomycin	*vanA*, *vanB*, *vanO*, *vanR*, *vanS*, *vanTG*, *vanWB*, *vanXYG*, *vanYG1*	59% [13,27,28,52,54,55,56,57,62, 63,64,66,68,69,71,72,76,77,78,80,84,85,86]	33% [27,52,54,56,57,62, 63,64,68,71,76,80,85]
Integron-integrase	-	-	*intI1*, *intI2*, *intI3*, *attI*, *qacEΔ1*	54% [21,27,28,55,56,57,60,63, 64,65,66,68,69,71,72,76,77,80,83,84,85]	21% [21,56,60,65,69,76,80,83]
Macrolides	Protein Synthesis Inhibition	Azithromycin, Carbomycin, Erythromyci	*ereA*, *ereB*, *ereD*, *ermA*, *ermB*, *ermC*, *ermM*, *ermML*, *ermQ*, *ermTR*, *lpeA*, *lpeB*, *mefA/E*, *mefE*, *mph*, *mphA*, *mphG*, *mphI*, *mphM*, *msrA/B*, *macA*, *macB*, *vatB*, *vatI*	69% [13,27,28,52,54,55,56,57,60,62, 63,64,66,68,70,71,72,75,76,77,78,79,80,83,84,85,86]	46% [27,52,54,60,62, 63,64,68,71,72,75,76,77,78,79,80,83,86]
Multidrug efflux systems	-	-	*abcA*, *acrA*, *acrE*, *abeM*, *abeS*, *acrB*, *adeA*, *adeB*, *adeC*, *adeF*, *adeG*, *adeH*, *adeI*, *adeJ*, *adeK*, *adeL*, *adeN*, *adeR*, *adeS*, *amvA*, *arlR*, *arlS*, *axyY*, *bcr*, *bepE*, *bmr*, *bmrA*, *carA*, *chrB*, *ceoB*, *clbB*, *cmeB*, *cpxA*, *cpxR*, *crp*, *emrA*, *emrB*, *emrE*, *emrK*, *emrY*, *ermZ*, *golS*, *lmrS*, *lsaC*, *marA*, *mdsB*, *mdtA*, *mdtB*, *mdtC*, *mdtE*, *mdtH*, *mdtK*, *mexA*, *mexB*, *mexD*, *mexF*, *mexH*, *mexI*, *mexK*, *mexQ*, *mexV*, *mexW*, *mtrA*, *muxB*, *muxC*, *norM*, *oleB*, *opmB*, *opmH*, *oprA*, *oprN*, *oqxA*, *oqxB*, *pmpM*, *poxtA*, *Qac*, *qacH*, *salA*, *smeB*, *smeE*, *smeR*, *smeS*, *srmB*, *stp*, *tla*, *tolC*, *vgaC*, *ybhR*, *yheI*	41% [27,28,55, 56,57,60,63,64,66,68,71,72,76,80,84,85]	33% [27,55,56,57,60,63,64,68,71, 72,76,80,84]
Nitrofuran	DNA Damage Agent	Nitrofurantoin	-	54% [27,28,52,54,55,56,57,58,62, 63,64,66,68,71,72,76,78,79,80,84,85]	13% [52,54,62,78,79]
Oxazolidinone	Protein Synthesis Inhibition	Linezolid	-	46% [27,28,54,55,56,57,62, 63,64,66,68,71,72,76,78,80,84,85]	5% [54,62]
Polymyxin	Cell Membrane Damage Agent	Colistin Sulphate (Polymyxin E)	*phoP*	49% [27,28,54,55,56,57,63,64,66,68,71,72,76,79, 80,81,83,84,85]	13% [54,68,79,81,83]
Rifamycins	Transcription Inhibition	Rifampicin, Rifamycin	*arr-1*, *rpoB*, *rpbA*, *rphA*, *mexF*, *iri*	49% [27,28,52,54,55,56,57,62, 63,64,65,67,68,71,72,76,79,80,84]	26% [27,52,54,62,68,71,72,76,79,84]
Sulfonamides	Folate Synthesis Inhibition	Sulfamethizole, Sulfamethoxazole, Sulfonamide	*sulI*, *sulII*, *sulIII*, *sulA*	82% [13,27,28,52,53,55,56,57,58,59,60,61,63,64,66, 67,68,70,71,72,73,74,75,76,77,79,80,81,82,83,84,85]	56% [13,21,52,53,58,59,60,61,67,68,70, 71,72,73,74,75,77,79,80,81,82,83]
Tetracyclines	Protein Synthesis Inhibition	Doxycycline, Minocycline, Tetracycline, Tigecycline	*otrA*, *otrB*, *tap*, *tet34*, *tet35*, *tet39*, *tet41*, *tet42*, *tet51*, *tetA*, *tetAP*, *tetB*, *tetBP*, *tetC*, *tetD*, *tetE*, *tetG*, *tetK*, *tetL*, *tetM*, *tetO*, *tetP*, *tetQ*, *tetR*, *tetS*, *tetW*, *tetX*, *tetZ*	90% [13,27,28,52,53,54,55,56,57,58,59,60,61,62,63,64,66,67,68,69,71, 72,73,74,76,77,78,79,80,81,82,83,84,85,86]	74% [27,28,52,53,54,55,56,57,58,59,60,61,62,64,67,68,71, 72,73,74,76,77,79,80,81,82,83,84,86]
Trimethoprim	Folate Synthesis Inhibition	Trimethoprim	*drfA1/6/7/17*	51% [27,28,52,55,56,57,59,61,63,64,66, 67,68,71,72,76,80,81,84,85]	26% [43,59,61,63,64,66,67,68,71,80]
Others	-	Acriflavin, Aminocoumarin, Bacitracin, Bicyclomycin, Clindamycin, Daptomycin, Doxorubicin, Fosmidomycin, Fusidic Acid, Lantibiotic, Lincomycin, Lincosamide, Mupirocin, Nitroimidazole, Polymycin, Puromycin, Streptothricin, Tetracenomycin, Triclosan	*alaS*, *arnA*, *bacA*, *bcr-1*, *drrA*, *floR*, *gyrB*, *ileS*, *lnuA*, *lsaA msbA*, *mupA*, *mupB*, *novA*, *parY*, *satA*, *trlC*, *ugd*	44% [27,28,54, 55,56,57,61,63,64,66,68,71,72,78,80, 84,85]	36% [27,54, 55,56,57,61,63,64,68,71,72,80,84,85]
